# Preparation, immunological and pharmacological effects of flavonoids in Scutellariae radix: a review

**DOI:** 10.3389/fphar.2025.1732322

**Published:** 2026-01-28

**Authors:** Haixia Chen, Yumin Wei, Jing Song, Yue Yang, Yanli Chen, Jiteng Sun, Daoming Bai, Zhiqiang Sun, Mingze Wu, Xiaomei Liu, Yanru Lin, Shaoping Wang, Long Dai, Yanan Li

**Affiliations:** 1 College of Pharmacy, Shandong University of Traditional Chinese Medicine, Jinan, China; 2 Institute of Pharmacy, Shandong University of Traditional Chinese Medicine, Jinan, China; 3 School of Pharmacy, Binzhou Medical University, Yantai, China; 4 Shandong Hanfang Pharmaceutical Co., Ltd., Jinan, China; 5 Shandong Yuze Pharmaceutical Industry Technology Research Institute Co., Ltd., Dezhou, China; 6 Shandong Key Laboratory of Digital Traditional Chinese Medicine, Shandong University of Traditional Chinese Medicine, Jinan, China

**Keywords:** flavonoids, immunological and pharmacological effects, *In vivo* metabolism, new dosage forms, Scutellariae radix, targeted therapy

## Abstract

In traditional Chinese medicine theory, Scutellaria baicalensis Georgi [Lamiaceae; Scutellariae radix] (SR) is bitter and cold in nature. It enters the lung, gallbladder, spleen, large intestine, and small intestine meridians. It clears heat and dries dampness, purges fire and detoxifies, stops bleeding, and stabilizes pregnancy. It excels at clearing lung fire and upper-body heat. Flavonoids, the primary active compound of SR, undergo metabolism *in vivo* through Phase I and Phase II reactions as well as intestinal flora-mediated processes. Modern pharmacological research indicates that flavonoid compounds exhibit diverse biological activities in immune modulation, antiviral, anti-inflammatory, antibacterial, and antitumor effects. In recent years, novel formulations such as nanomedicines and liposomes have garnered increasing attention to enhance their stability and bioavailability. This review systematically summarizes the research progress on flavonoid compounds in SR, comprehensively elaborating on their phytochemistry, extraction methods, separation and purification techniques, *in vivo* metabolism, immunological and pharmacological effects, toxicity, and novel dosage forms. It provides theoretical foundations and practical references for the further research, development, and rational application of these compounds.

## 1 Introduction

Traditional medicine, as a vital component of global health systems, serves approximately 80% of the world’s population for disease prevention and treatment. Through extensive clinical practice, it has developed a rich system for utilizing medicinal plants (Yunusoglu et al., 2025). *Scutellaria baicalensis* Georgi, a member of the Lamiaceae family, is renowned for its dried root, SR. Flavonoids are its primary active components and possess numerous medicinal values. In China, SR is predominantly cultivated in Inner Mongolia, Hebei, Gansu, Shanxi, Shandong, Heilongjiang, and other regions. As a commonly used medicinal herb, it is also grown in various parts of the world, including Russia, East Siberia, Mongolia, Korea, and Japan (Zhao et al., 2019). SR was first documented in the *Shennong Herbal Classic* (*Shennong Bencao Jing*) during the Eastern Han Dynasty. Known as ‘Huang Qin’ in traditional Chinese medicine, it has been used for over 2000 years (Wang Z. L. et al., 2018). SR is officially recognized in the *Pharmacopoeia of the People’s Republic of China* (*Chinese Pharmacopoeia*), as well as in the *European Pharmacopoeia* and the *British Pharmacopoeia*. Based on traditional Chinese medicine theory, SR is associated with the meridians of the lung, gallbladder, spleen, large intestine, and small intestine. After stir-frying with wine, the medicine was introduced upward, and the upper energizer heat was cleared (Cui et al., 2017).

**FIGURE 1:**
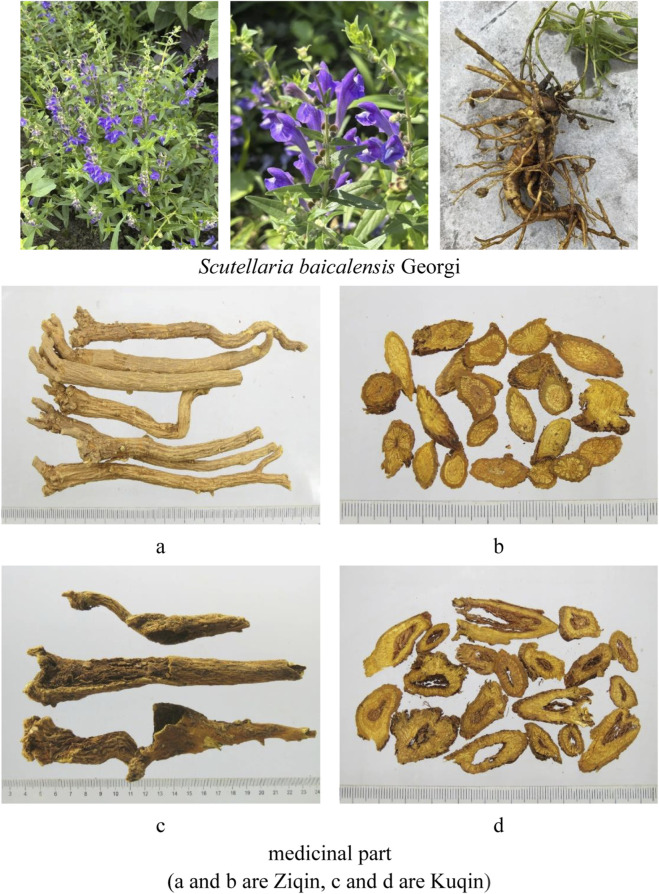
The original plants and decoction pieces of SR. (**(a,b)** are Ziqin, **(c,d)** are Kuqin).

In ancient literature, SR is categorized into two distinct specifications: Kuqin and Ziqin, each with its unique applications. Tao Hongjing, in his *Annotations to the Classic of Materia Medica* (*Bencao Jingji Zhu*), noted: ‘The round variety, known as Ziqin, is esteemed, while the broken one, Suqin, with a decayed interior, is termed Fuchang; however, the darker and more solid forms are preferred.’ Kuqin, an aged root harvested after 3 years, is hollow and withered, known for its efficacy in clearing upper Jiao lung fire; whereas Ziqin, the seed root harvested within 2 years, the root is dense and solid, and it is good at clearing damp heat in the large intestine. Currently, its separate use in clinical practice has been discontinued, with SR decoction pieces being exclusively utilized for medicinal purposes (Zhang F. L. et al., 2020). SR is widely used in classical prescriptions of traditional Chinese medicine, and is generally used to treat respiratory diseases, such as Xiaochaihu Decoction (Cheng et al., 2023), Dayuan Decoction (Guo et al., 2023), Huanglian Jiedu Decoction (Chen et al., 2022) and so on. A large number of clinical results have shown that SR has a good effect on severe acute respiratory syndrome coronavirus 2 (SARS-CoV-2) caused by coronavirus disease 2019 (COVID-19) (Song J. et al., 2021; Song et al., 2020) (Figure 1).

Over recent decades, a variety of compounds have been isolated from SR, including flavonoids, volatile oils, terpenoids, polysaccharides, β-sitosterol, campesterol, and stigmasterol (Zhao et al., 2019). In recent years, flavonoids have attracted much attention due to their extensive biological activities such as immunomodulation, antivirus, anti-inflammatory, antibacterial, hypolipidemic, and antitumor (Pietta, 2000; Serafini et al., 2010; Farhadi et al., 2019; Umar et al., 2015; Sun et al., 2022). Flavonoids are natural plant compounds widely distributed throughout all parts of plants, including roots, stems, leaves, flowers, and fruits. They constitute approximately 60% of total natural polyphenols and serve as key substances enabling plants to perform defensive functions and exhibit diverse biological activities (Yunusoğlu et al., 2025). They are also commonly incorporated into our daily diets, including in fruits (Ahmed and Eun, 2018), vegetables (Toh et al., 2013), and tea (Hodgson and Croft, 2010), all of which are abundant in flavonoids.

Despite demonstrating significant potential in preclinical studies, flavonoids face critical challenges in achieving effective translation to clinical applications. Many bioactive flavonoid components suffer from poor water solubility, low intestinal absorption rates, and rapid metabolism, severely limiting their therapeutic efficacy and hindering new drug development. Notably, these pharmacokinetic challenges are often closely linked to their specific chemical structures—such as hydroxyl substitution patterns and glycosylation patterns—which determine their biological activity but also impact solubility, stability, and *in vivo* processes. Therefore, overcoming these bottlenecks through novel drug delivery systems has become a key research focus.

This review synthesizes the current research on the phytochemistry, extraction methods, separation and purification methods, metabolism *in vivo*, immunological and pharmacological effects, toxicity and new dosage forms of flavonoids in SR, with the aim of advancing understanding and offering new perspectives for its development, pharmacodynamic material basis interpretation, and clinical application.

## 2 Methods

This study conducted a comprehensive search of journal articles, books, and theses from databases including Web of Science, PubMed, Google Scholar, Elsevier, ACS Publications, Wiley Online Library, Springer Link, and CNKI. The literature search was primarily limited to publications from the last 5 years; however, a small number of earlier seminal studies critical to this review were also included. Using SR and its flavonoid components as search subjects, the literature was retrieved using keywords such as phytochemistry, extraction and separation, *in vivo* metabolism, pharmacological activity, toxicology, or novel formulations as keywords. Relevant literature was selected based on publication types such as original research papers or high-quality reviews. Studies on flavonoids not derived from SR, mixed extracts of flavonoids with other components from SR, and literature lacking temporal relevance were excluded. All retrieved documents underwent comprehensive review to ensure inclusion of pertinent materials. Furthermore, detailed information regarding SR was obtained from relevant monographs such as the *Chinese Pharmacopoeia*. Visual analysis of phytochemical constituents was performed using ChemDraw 21.0 software.

## 3 Phytochemistry

### 3.1 Chemical composition

The flavonoids in SR are mainly flavonoid aglycones and their glycosides, and a few are flavonoid alcohols, dihydroflavones and other derivatives. The core of the structure is the flavonoid nucleus of C6-C3-C6, and most of the components have hydroxyl or methoxy substitution in the A ring and B ring. Some components combine with glucuronic acid to form glycosides through 7-hydroxyl. At present, more than 100 kinds of flavonoid aglycones and their glycosides have been successfully isolated and identified from a variety of SR (Li Y. et al., 2023). The most widely studied representative components are shown in Table 1, especially baicalin, baicalein, wogonoside and wogonin, which constitute the four flavonoids with the most pharmacological significance in SR. Baicalin is one of the most abundant flavonoids in SR, which is an index component of quality control of SR clearly recorded in *Chinese Pharmacopoeia* (National Pharmaco poeia Commission, 2020). Its molecular formula is C_21_H_18_O_11_, molecular weight is 446.37, and it is a pale-yellow powder at room temperature. Baicalin, a flavonoid derivative with a glucuronic acid structure, undergoes hydrolysis to produce baicalein and glucuronic acid. Because the flavonoid aglycone and glucuronic acid in the structural formula of baicalin can form intramolecular hydrogen bonds, the solubility of baicalin in water is extremely low. Modern pharmacological studies have shown that flavonoids in SR have anti-inflammatory, antioxidant, anti-tumor, antiviral, antibacterial and other effects (Wang X. et al., 2022; Hu et al., 2022; Singh et al., 2021; Russo et al., 2020; Bao et al., 2022), which provides an important basis for the wide application of SR in the field of traditional Chinese medicine.

**TABLE 1:** Representative components of SR.

Compound	Molecular formula	Structural formula	References
Baicalin	C_21_H_18_O_11_	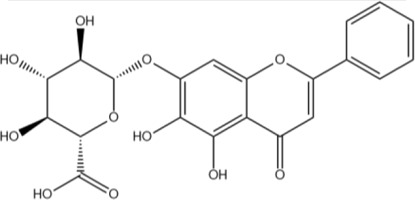	Li et al. (2023a)
Baicalein	C_15_H_10_O_5_	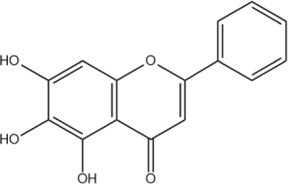	Li et al. (2023a)
Wogonoside	C_22_H_20_O_11_	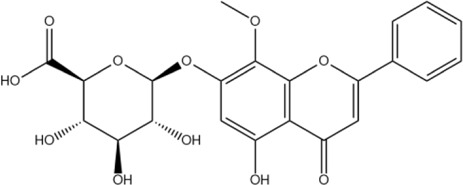	Li et al. (2023a)
Wogonin	C_16_H_12_O_5_	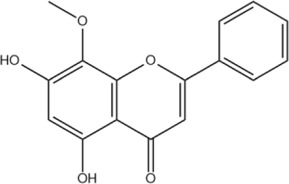	Li et al. (2023a)
Scutellarin	C_21_H_18_O_12_	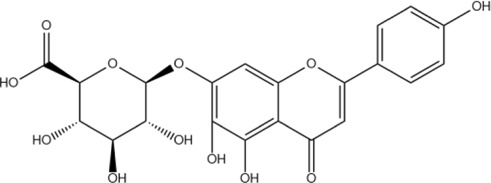	Li et al. (2023a)
Scutellarein	C_15_H_10_O_6_	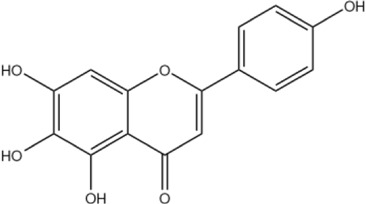	Chen et al. (2024)
Chrysin	C_15_H_10_O_4_	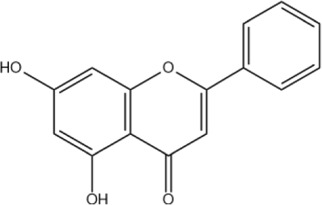	Chen et al. (2024)
Oroxylin A	C_16_H_12_O_5_	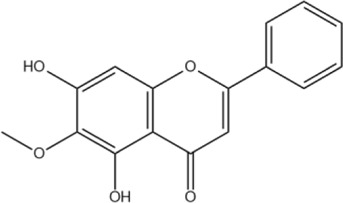	Li et al. (2023a)
Apigenin	C_15_H_10_O_5_	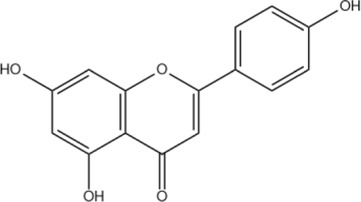	Li et al. (2023a)
Luteolin	C_15_H_10_O_6_	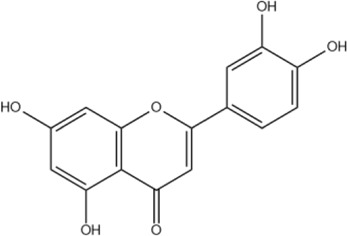	Li et al. (2023a)
Negletein	C_16_H_12_O_5_	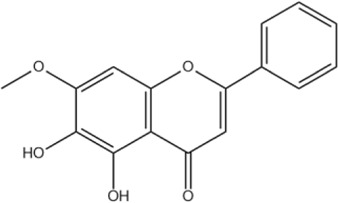	Chen et al. (2024)
4′-Hydroxywogonin	C_16_H_12_O_6_	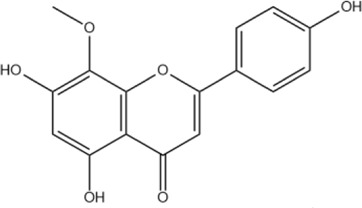	Gao et al. (2025)
7-O-methylwogonin	C_17_H_14_O_5_	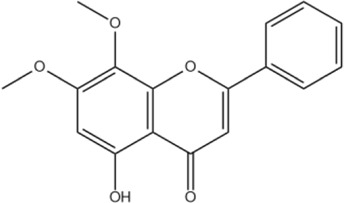	Gao et al. (2025)
Oroxin B	C_27_H_30_O_15_	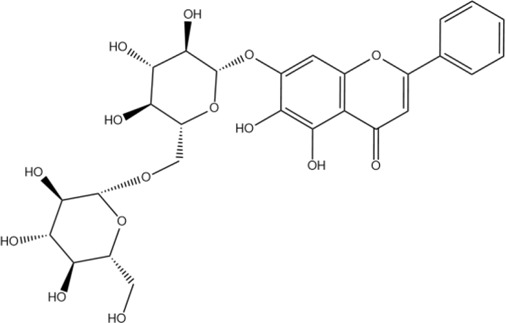	Gao et al. (2025)
Apigenin-7-O-β-D-glucopyranoside	C_21_H_20_O_10_	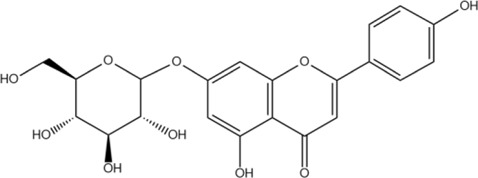	Li et al. (2023b)
4′,5,7,8-tetrahydroxyflavanone	C_15_H_12_O_6_	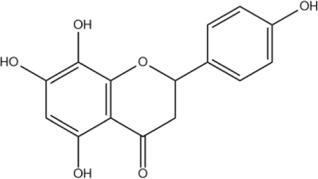	Chen et al. (2024)
Chrysin-7-O-Beta-D-glucoronid	C_21_H_18_O_10_	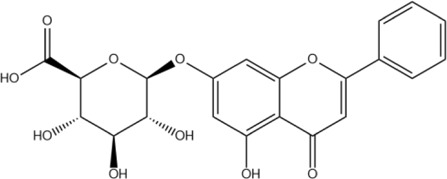	Li et al. (2023b)

### 3.2 Source

Some flavonoids in SR are not only present in SR but are also found in *Oroxylum indicum* (L.) Kurz [Bignoniaceae; Oroxyli semen] (*O. indicum*). Fuentes et al. (2015) isolated six flavonoids, including baicalin, baicalein, Oroxylin A and apigenin, from the methanol extract of *O. indicum*. Rojsanga et al. (2020) quantitatively analyzed the content of three primary flavonoids in *O. indicum* using high-performance liquid chromatography (HPLC), revealing that baicalin was the predominant compound among them. The seed extract exhibited the highest baicalin content, constituting 24.24%, followed by the third-week shoot extract of tissue-cultured seedlings, which contained 14.78% baicalin. Rojsanga et al. (2023) also utilized HPLC to quantify the extracts from young fruits, mature green fruits, dry pod peels, and seeds of *O. indicum*. The findings confirmed baicalin as the primary compound in all extracts, with concentrations ranging from 0.4% to 11%. Sithisarn et al. (2019) demonstrated that flavonoids, including baicalein, baicalin, and chrysin, in *O. indicum* possess antibacterial properties. Furthermore, *O. indicum* seed extracts containing baicalin and baicalein have been investigated for their potential in treating major depressive disorder (Chalermwongkul et al., 2023).

Nevertheless, the content of flavonoids in *O. indicum* is relatively low, necessitating larger quantities of raw materials, solvents, and energy to obtain equivalent amounts of flavonoids. Additionally, *O. indicum* contains significant amounts of polysaccharides, flavonoids, volatile oils, and other compounds, which complicates impurity separation and increases production costs. In contrast, the extraction of flavonoids from SR roots is far more efficient, leading to *O. indium*’s lower industrial extraction value. Consequently, the extraction and purification of flavonoids from SR are widely adopted.

## 4 Extraction methods

Flavonoids refer to a series of compounds in which two benzene rings are connected by three carbon atoms, which are widely found in green plants and daily foods (Wen et al., 2021). The extraction methods of flavonoids include extraction method, steam distillation method, ultrasonic assisted extraction method, microwave assisted extraction method, supercritical fluid extraction method and solvent extraction method, *etc.* (Rodríguez De Luna et al., 2020; Cui et al., 2022; Stalikas, 2007; Dias et al., 2021). Each method offers unique advantages, and in some cases, combining multiple techniques can further improve extraction efficiency (Xie et al., 2014).

### 4.1 Solvent extraction process

#### 4.1.1 Water decoction extraction method

Water decoction extraction method is a common extraction method. In the 2020 edition of *Chinese Pharmacopoeia*, SR extract is extracted by hot water decoction method (National Pharmaco poeia Commission, 2020). Cao et al. (2025) used the total evaluation normalization combined with response surface method to optimize the process parameters of SR water extract, and finally increased the extraction rates of baicalin and total flavonoids to 11.97% and 27.03%, respectively. In this study, the single factor combined with orthogonal test design method was utilized to systematically investigate the key influencing factors, such as soaking conditions, solvent dosage, extraction time, and extraction times, on the extraction process of baicalin. The optimization of these factors was evaluated based on the extraction rate and content of baicalin, aiming to establish an efficient and reliable preparation method. Finally, the medicinal materials were extracted twice using boiling water, with the first extraction lasting 60 min and the second 30 min, each using 10 + 5 times the amount of the material. Under these optimized conditions, the extraction rate of baicalin reached 15.08% (Gao et al., 2021). Zhai and Zhao (2020) obtained the optimum water extraction process of baicalin in SR by response surface method: extraction time 2.25h, material-liquid ratio 56.52 mL/g, extraction times 2.12 times. Under this condition, the extraction rate of baicalin was 17.10%. Considering the needs of practical operation, the extraction time can be adjusted to 2.25h, material-liquid ratio 57 mL/g, extraction times 2 times.

The water decoction extraction method boasts simplicity in operation and equipment, low production costs, environmental friendliness, and a high flavonoids extraction rate, rendering it suitable for industrial production and possessing significant practical value.

#### 4.1.2 Alcohol extraction method

The alcohol extraction method serves as a means to isolate and extract components of traditional Chinese medicine, leveraging the solubility properties of methanol and ethanol as solvents. A single factor experiment was used to study the effects of ethanol concentration, solid-liquid ratio, soaking time, extraction temperature and extraction time on the extraction rate of total flavonoids from SR. Then, the response surface test was used to optimize the extraction process of total flavonoids, and the optimal process conditions were predicted by establishing a mathematical model (Yan et al., 2023). Jin et al. (2021) used the yield of baicalin as the index, and the response surface method was used to investigate the effects of ethanol concentration, liquid-material ratio and extraction time on the yield of baicalin. The optimal conditions for the extraction of baicalin comprised refluxing in 77% ethanol solvent, with a specified liquid-to-material ratio of 10:1 for 2 h and extracted twice. Under these conditions, the extraction rate of baicalin was 12.55%. Sun et al. (2016) determined the methanol extraction process of baicalin by orthogonal experiment: 70% methanol was refluxed and extracted three times at 80 °C, the ratio of material to liquid was 1:8,1:6,1:4, and the time was 2h, 2h, 1h, respectively. The extraction rate of baicalin was 6.96%. The cost of alcohol extraction method is high and the safety is poor, so it is relatively less used in industrial production.

### 4.2 Ultrasonic extraction method

Ultrasonic extraction method can make use of the cavitation effect of ultrasonic wave. The burst of tiny bubbles in ultrasonic cavitation will produce great pressure, so that the rupture of plant cell wall and the whole organism can be completed in an instant, which shortens the crushing time. At the same time, the vibration effect generated by ultrasonic wave strengthens the release, diffusion and dissolution of intracellular substances, thus significantly improving the extraction efficiency. At the same time, it also avoids the destruction of active components by high temperature. The application of ultrasonic extraction technology in the extraction of active components from medicinal plants has shown great potential and advantages, with its efficiency, precision, and environmental friendliness making it a significant tool in the modernization and standardization of traditional Chinese medicine (Yusoff et al., 2022).

Li et al. (2020) used the central composite design-response surface methodology to investigate the effects of ethanol concentration, liquid-to-material ratio, and extraction time on the yield of baicalin during ultrasonic extraction. The results indicated that the optimal extraction rate of baicalin was 8.32%, achieved through ultrasonic extraction with an ethanol concentration of 77%, a liquid-to-material ratio of 22:1, an extraction duration of 52 min, and a single extraction process. In order to optimize the best ultrasonic extraction process of baicalin, Liu et al. (2017) used orthogonal experimental design to study the effects of four factors, such as material-liquid ratio, ultrasonic power, ethanol concentration and pH value, on the ultrasonic extraction process of baicalin with the yield of baicalin as the index. The results showed that the effects of various factors on the yield of baicalin from large to small were ethanol concentration, material-liquid ratio, pH value and ultrasonic power. The optimal conditions for ultrasonic extraction of baicalin were ethanol concentration of 55%, material-liquid ratio of 1:18, pH value of 4.0, and ultrasonic power of 59 kHz. Under these conditions, the extraction rate of baicalin extract could reach 16.52%. Huang et al. (2024) used baicalin, wogonoside, baicalein and wogonin as evaluation indexes, and ethanol concentration, ultrasonic time, ultrasonic temperature and solid-liquid ratio as investigation factors. On the basis of orthogonal test, analytic hierarchy process, entropy weight method and analytic hierarchy process-entropy weight method were used to determine the weight coefficient of each index, and the extraction process of flavonoids in SR was optimized. Ultrasonic extraction method has the advantages of low solvent consumption, short operation time, low extraction temperature, low energy consumption, high yield, short extraction time and high extraction efficiency (Shen et al., 2023). It is often used for the extraction of various components in traditional Chinese medicine.

### 4.3 Microwave extraction method

The microwave extraction method induces thermal stress within cells through molecular dipole rotation and the significant energy present in the microwave field, leading to cell rupture, enhanced solvation of the sample, and accelerated extraction of the target compound (Mirzadeh et al., 2020). It also overcomes the overheating issues common in traditional heating methods, reduces solvent usage, shortens extraction time, and improves extraction efficiency (Ferrara et al., 2023). Fu et al. (2010) examined six process parameters, including solvent type, extraction temperature, soaking time, holding time, material-to-liquid ratio, and extraction times. An orthogonal experiment was then conducted, identifying the optimal microwave extraction conditions: 30% ethanol, a material-to-liquid ratio of 1:8, soaking for 30 min, microwave heating at 120 °C for 10 min, followed by two extraction cycles. Under these conditions, the baicalin extraction yield reached 8.92%. Han et al. (2011) optimized the microwave extraction process for baicalin, using the baicalin content as an index. The optimal extraction conditions were determined to be a material-to-liquid ratio of 1:15, 70% ethanol, an extraction duration of 5 min, and an extraction temperature of 90 °C, resulting in an average baicalin extraction rate of 18.79%. Compared to traditional water extraction methods, microwave extraction offers significant advantages, including reduced time, higher efficiency, and energy conservation.

### 4.4 Supercritical fluid extraction method

Supercritical fluid extraction method is a technique for extracting target components from solids or liquids using supercritical fluids as solvents. It comprises two stages: extraction and separation, which can be performed under dynamic or static conditions. Carbon dioxide, due to its low critical temperature and pressure, as well as its chemical inertness, has emerged as the most commonly used supercritical extractant (Uwineza and Waśkiewicz, 2020). Supercritical fluid extraction offers several advantages, including short extraction times, suitability for volatile compounds, high extraction efficiency, minimal solvent usage, and no environmental pollution (Yousefi et al., 2021). However, due to carbon dioxide’s limited solubility for certain polar compounds, small amounts of modifiers such as methanol, ethanol, or water are often added to enhance the solubility of polar substances and improve extraction efficiency. Chang et al. (2007) explored the effects of modifiers, temperature, pressure, and dynamic extraction time on the yield of baicalin using an orthogonal design. The results showed that the choice of modifier was the most significant factor affecting the extraction rate of baicalin. While extraction temperature of 40 °C–60 °C, extraction time of 30–90 min, and extraction pressure of 200–400bar all influenced the extraction, their impact was less pronounced than that of the modifier. The optimal extraction conditions for baicalin were found to be a temperature of 60 °C, an extraction time of 60 min, and an extraction pressure of 200 bar, with 1,2-propanediol as the modifier. Under these conditions, the extraction rate of baicalin was 8.16% ± 0.62%. In comparison, the baicalin extraction using 70% methanol-modified supercritical CO_2_ resulted in a yield of 7.82% ± 0.59%, which was slightly higher than the 7.66% ± 0.63% obtained using the Soxhlet extraction method (with 70% methanol and an extraction time of 7 h).

Compared to organic solvent extraction, supercritical fluid extraction significantly reduces extraction time and solvent consumption, while also eliminating the challenges of solvent recovery and residue. It holds promise as an effective method for extracting active components from Chinese medicinal materials.

### 4.5 Deep eutectic solvent extraction method

Deep eutectic solvents are mainly composed of hydrogen bond donors such as polyols, urea and carboxylic acids and hydrogen bond acceptors such as quaternary ammonium salts (such as choline chloride). When two or more different hydrogen bond acceptors and hydrogen bond donors are mixed at a specific molar ratio and temperature (typically 60 °C), a uniform, transparent liquid is formed, known as a deep eutectic solvent (Liu et al., 2022). In 2003, the Abbott team pioneered the preparation of deep eutectic solvents using choline chloride and urea as raw materials (Abbott et al., 2003). Deep eutectic solvents offer advantages over ionic liquids, including simpler, faster, and more cost-effective preparation, along with lower toxicity. As a result, they have gained recognition as nearly non-toxic green reagents and are increasingly used in the separation of natural compounds. Some researchers have also explored the use of Deep eutectic solvents for extraction purposes (Mushtaq et al., 2024).

A green and environmentally friendly switching polar supramolecular deep eutectic solvent was prepared for the extraction of flavonoids in SR. The optimal extraction process parameters were determined by single factor experiment and response surface method (Zhang, 2025). Oomen et al. (2020) studied the ability of natural deep eutectic solvents to extract flavonoids from SR as a substitute for traditional organic solvents such as methanol aqueous solution or ethanol aqueous solution. The results showed that the extraction rate of baicalein, scutellarein, wogonin, oroxylin A was 2–6 times higher than that of methanol aqueous solution, and the extraction rate of baicalin, wogonoside and oroxyloside was 1.5–1.8 times higher than that of methanol aqueous solution. Wang H. et al. (2018) combined deep eutectic solvents with ultra-high pressure extraction to isolate baicalin from SR, and finally the maximum extraction rate of baicalin reached 11.68%, which was significantly higher than the traditional extraction method. Scanning electron microscopy of SR showed that the dissolution of chemical components in the root tissue was enhanced with the combination of deep eutectic solvent and ultra-high pressure extraction. This suggests that the combination of these two methods is a fast and efficient means for extracting baicalin from SR. Hao et al. (2020) used ultrasound-assisted deep eutectic solvent method to extract flavonoids from SR, so that the content of flavonoids reached the highest. Compared to organic solvent extraction, deep eutectic solvents extraction increased the flavonoids yield. However, large-scale application remains challenging due to difficulties in recovery and regeneration of the solvents.

Currently, water decoction extraction is the most commonly used and simplest method for extracting flavonoids. The deep eutectic solvent extraction method, a relatively recent development, offers a promising alternative. In summary, while the water decoction extraction method is straightforward and practical, it is plagued by lengthy extraction times and cumbersome operational procedures. Compared with the solvent extraction method, the ultrasonic-assisted extraction method, microwave-assisted extraction method, supercritical fluid extraction method, while the deep eutectic solvent extraction method can extract flavonoids more swiftly, its extraction efficiency lags behind the solvent extraction method. Consequently, water decoction extraction remains the preferred method. Other techniques, such as electromagnetic cracking extraction (Wang R. G. et al., 2019), infrared extraction (Li et al., 2017), semi-bionic extraction (Jiang et al., 2011), and flash extraction (Wang et al., 2014), also offer potential for the extraction of flavonoids but require further investigation (Table 2).

$$\text{Flavonoids extraction rate }\left(\%\right)=\text{Flavonoids quality }\left(g\right)/\text{SR quality }\left(g\right)\times 100\%$$

**TABLE 2:** Extraction methods of flavonoids.

Extraction method		Extraction conditions	Extraction rate	References
Solvent extraction process	Water decoction extraction method	Raw material for extraction: SR medicinal herb; extraction times: 3 times; extraction time: 2.5 h; material-liquid ratio: 1:17.	Baicalin 11.97% Total flavonoids 27.03%	Cao et al. (2025)
		Raw material for extraction: SR root; extraction times: 2 times; extraction time: 1 h, 2 h; material-liquid ratio: 1:10 and 1:5.	Baicalin 15.08%	Gao et al. (2021)
		Raw material for extraction: SR medicinal herb; extraction times: 2.12 times; extraction time: 2.25 h; material-liquid ratio: 1:56.52.	Baicalin 17.10%	Zhai and Zhao (2020)
	Ethanol extraction method	Raw material for extraction: SR decoction pieces; extraction solvent: 58.5% ethanol; soaking time: 45min; extraction temperature: 65.5 °C; extraction time: 2 h; material-liquid ratio: 1:47.	Total flavonoids 10.52%	Yan et al. (2023)
		Raw material for extraction: SR decoction pieces; extraction solvent: 77% ethanol; extraction times: 2 times; extraction time: 2 h; material-liquid ratio: 1:10.	Baicalin 12.55%	Jin et al. (2021)
	Methanol extraction method	Raw material for extraction: dried root of SR; extraction solvent: 70% methanol; extraction times: 3 times; extraction time: 2 h, 2 h, 1 h; material-liquid ratio: 1:8, 1:6, 1:4.	Baicalin 6.96%	Sun et al. (2016)
Ultrasonic extraction method		Raw material for extraction: SR decoction pieces; extraction solvent: 77% ethanol; extraction times: 1 time; ultrasonic time: 52 min; material-liquid ratio: 1:22.	Baicalin 8.32%	Li et al. (2020)
		Raw material for extraction: SR decoction pieces; extraction solvent: 55% ethanol; ultrasonic power: 59kHz; pH: 4.0; material-liquid ratio: 1:18.	Baicalin 16.52%	Liu et al. (2017)
		Raw material for extraction: SR medicinal herb; extraction solvent: 55% ethanol; ultrasonic time: 25 min; ultrasonic temperature: 60 °C; material-liquid ratio: 1:25.	The total content of baicalin, wogonoside, baicalein and wogonin 21.63%	Huang et al. (2024)
Microwave extraction method		Raw material for extraction: SR decoction pieces; extraction solvent: 30% ethanol; soaking time: 30 min; extraction time: 10 min; extraction times: 2 times; temperature: 120 °C; material-liquid ratio: 1:8.	Baicalin 8.92%	Fu et al. (2010)
		Raw material for extraction: dried root of SR; extraction solvent: 70% ethanol; extraction time: 5 min; extraction times: 1 time; extraction temperature: 90 °C; material-liquid ratio: 1:15.	Baicalin 18.79%	Han et al. (2011)
Supercritical fluid extraction method		Raw material for extraction: SR root; modifier: 1,2-propanediol; extraction temperature: 60 °C; extraction time: 60 min; extraction pressure: 200bar.	Baicalin 8.16%	Chang et al. (2007)
		Raw material for extraction: SR root; modifier: 70% methanol; extraction temperature: 60 °C; extraction time: 60 min; extraction pressure: 200bar.	Baicalin 7.82%	Chang et al. (2007)
Deep eutectic solvent extraction method		Raw material for extraction: SR medicinal herb; deep eutectic solvent: N, N-dimethylbenzylamine: octanoic acid, molar ratio: 1:2; moisture content: 30%; β-cyclodextrin content: 5.5wt%; material-liquid ratio: 1:60.	Baicalin, wogonoside, baicalein 15.80%	Zhang (2025)
		Raw material for extraction: dried root of SR; deep eutectic solvent: citric acid: β-alanine, molar ratio: 1:1; moisture content: 40%; extraction temperature: 40 °C; extraction time: 1 h; material-liquid ratio: 1:50.	baicalin 3.94% Scutellarein 0.75%	Oomen et al. (2020)
		Raw material for extraction: dried root of SR; deep eutectic solvent: citric acid: proline, molar ratio: 1:1; moisture content: 50%; extraction temperature: 40 °C; extraction time: 1 h; material-liquid ratio: 1:50.	Wogonin 1.94%	Oomen et al. (2020)
		Raw material for extraction: dried root of SR; deep eutectic solvent: citric acid: proline, molar ratio: 1:1; moisture content: 60%; extraction temperature: 40 °C; extraction time: 1 h; material-liquid ratio: 1:50.	Baicalein 0.32% Wogonoside 8.24% Oroxylin A 0.70% Oroxyloside 1.20%	Oomen et al. (2020)
		Raw material for extraction: dried root of SR; deep eutectic solvent: choline chloride: lactic acid, molar ratio: 1:1; moisture content: 40%; extraction pressure: 400MPa; extraction time: 4 min; material-liquid ratio: 1:110.	Baicalin 11.68%	Wang H. et al. (2018)
		Raw material for extraction: SR; deep eutectic solvent: betaine: acetic acid, molar ratio: 1:4; moisture content: 40%; extraction temperature: 52 °C; extraction time: 23 min; material-liquid ratio: 1:100.	Scutellarin 0.73% Baicalin 11.93% Baicalein 2.57% Wogonoside 1.26% Wogonin 0.41% Oroxylin A 0.17%	Hao et al. (2020)

## 5 Separation and purification methods

As the principal active ingredient in SR, the purity of flavonoids after separation and purification directly influences subsequent pharmacological studies and industrial production. The crude products of SR obtained from preliminary extraction and separation processes requires purification to remove impurities such as sugars, proteins, and pigments. Common purification techniques include acid precipitation, ultrafiltration, and chromatography. Below, the separation and purification methods, along with their respective advantages, disadvantages, and the purity of flavonoids in SR, are discussed.

### 5.1 Acid precipitation method

Baicalin is a glucuronide with weak acidity, making it soluble in alkaline solutions. Upon acidification, baicalin precipitates out, particularly in the presence of strong inorganic acids. This solubility difference between alkali and acid is exploited for purification, allowing baicalin to be separated from acidic impurities.

Chen (2018) employed water extraction combined with acid precipitation for baicalin extraction. The process involved boiling the material three times with water-2 h for the first extraction, and 1 h for the second and third extractions. The decoction was combined for three times and filtered. The filtrate was concentrated to a density of 1.03–1.08 (80 °C). The pH value was adjusted to 1.0–2.0 with 2 mol/L hydrochloric acid at 80 °C, kept for 1h, and stood for 24 h. After filtration, the precipitate was washed with water to pH 5.0, followed by washing with 70% ethanol to pH 7.0, and finally dried at low temperature. The resulting baicalin purity was 72.77%. Zhang (2012) studied the effects of holding temperature, holding time, pH, and standing time on acid precipitation using an orthogonal test. The optimal conditions were acid precipitation at pH 1.0–1.5, holding temperature at 80 °C, holding time of 120 min, and a standing time of 1 h, resulting in a baicalin purity of 80.32%. Li (2011) conducted an orthogonal test considering pH, holding temperature, and holding time, optimizing acid precipitation at pH 1.0–2.0, a temperature of 80 °C, holding time of 1 h, and standing for 12 h, achieving a baicalin purity of 67.22%. This method is advantageous due to its simplicity, rapid processing, low cost, stable performance, and good reproducibility, offering valuable insights for the industrial production of baicalin.

### 5.2 Ultrafiltration method

Ultrafiltration is a membrane separation technique that utilizes the microporous structure of semi-permeable membranes to apply a certain pressure, facilitating the separation or concentration of solutes with varying molecular weights in a solution (Kammakakam and Lai, 2023). Wang et al. (2007) employed a two-step ultrafiltration process, first using a 100K ultrafiltration column followed by a 5K ultrafiltration column, and then subjected the ultrafiltrate to acid precipitation. The acid precipitation conditions were as follows: 37% hydrochloric acid was added to adjust the pH to 3–4. After being placed for 1h, the filtrate was filtered, and then the pH was then adjusted to 1.5–2 at 40 °C, kept at 80 °C for 30 min, and allowed to stand for 12–16 h. The purity of baicalin after acid precipitation was 89.98%, which was superior to the conventional decoction method (where the purity of baicalin was 77.77%). Li (2011) also used a 100K ultrafiltration column and followed similar acid precipitation conditions: pH 1–2, 80 °C, 1 h of heat preservation, and standing for 12 h, resulting in a baicalin purity of 91.47%. Ultrafiltration offers several advantages, including a simple production process, short cycle time, operation at room temperature, energy savings, environmental friendliness, and high purification efficiency.

### 5.3 Chromatography method

#### 5.3.1 Resin absorption method

The resin adsorption method utilizes the ion exchange, adsorption, and chelation properties of resins to achieve selective adsorption, mainly for the separation, purification, and impurity removal of effective components in traditional Chinese medicine. A study investigated the effects of sample solution concentration, loading amount, washing volume, eluent (ethanol) volume fraction, and elution volume as influencing factors. Four macroporous adsorption resins (AB-8, HPD300, NKA-9, SP825) were screened using static and dynamic adsorption tests, and the purification process for flavonoid components in SR was optimized (Li and Liu, 2017). Du et al. (2012) studied the separation and purification of baicalin and wogonoside from SR extract using the non-polar resin HPD-100. The optimized process involved an equilibrium time of 180 min, pH 5, sample volume of 2BV/h, adsorption at 25 °C, and elution with a 20%–40% ethanol aqueous solution. The contents of baicalin and wogonoside were increased by 3.6 times and 12.0 times, respectively, and the recovery rates were 85.70% and 65.60%, respectively. These results demonstrated that baicalin and wogonoside can be efficiently and conveniently prepared and separated through the adsorption and desorption of HPD-100 resin. Li (2011) used a D104 macroporous resin column for the separation and purification of SR’s crude extract. After three rounds of adsorption, the extraction was left to stand for 14 h, eluted with 70% ethanol, and acid precipitation was performed. The resulting baicalin purity was 93.42%.

The resin adsorption method offers high extraction rates, high flavonoids content, low cost, and ease of scaling up for industrial production. However, the treatment of the resin can be challenging and may result in product contamination.

#### 5.3.2 Counter-current chromatography method

Counter-current chromatography is a liquid-liquid chromatography technique that separates components based on their partitioning between two immiscible solvents. In this method, components are separated by varying partition coefficients as they pass through two solvent phases, eliminating the need for a solid support. Wu et al. (2005) established a method for the separation and purification of baicalin and wogonoside in SR by high-speed counter-current chromatography. Using an ethyl acetate-methanol-1% acetic acid water (5:0.5:5) two-phase solvent system, 58.1 mg of baicalin with a purity of 99.20% and 17.0 mg of wogonoside with a purity of 99.00% were isolated from 120 mg of crude SR. The structures of baicalin and wogonoside confirmed using ^1^H NMR and ^13^C NMR. Wang C. et al. (2019) observed that baicalin and wogonoside exhibited poor solubility in non-polar solvents, making it difficult to separate using conventional counter-current chromatography. Therefore, baicalin and wogonoside were separated and purified from the extract of SR by pH zone countercurrent chromatography and conventional countercurrent chromatography. In the pH-zone-refining method, an environmentally friendly two-phase solvent system of n-butanol-ethyl acetate-water (2:3:5) was used, with trifluoroacetic acid added to the organic phase to a final concentration of 10 mmol L^−1^, and ammonia was added to the aqueous phase to a final concentration of 10 mmol L^−1^. Conventional counter-current chromatography utilized an ethyl acetate-ethanol-3 mmol/L hydrochloric acid (10:1:10) two-phase solvent system. Initially, 186.7 mg of baicalin with a purity of 95.30% and a mixture of 143.4 mg of baicalin and wogonoside were obtained from 500 mg of crude SR extract using pH-zone-refining counter-current chromatography. A secondary separation with conventional counter-current chromatography yielded 64.3 mg of baicalin with a purity of 98.20% and 46.1 mg of wogonoside with a purity of 98.9%. This method proved efficient in producing high purity and quantity of baicalin and wogonoside in a relatively short period. Wang C. et al. (2020) further optimized the separation of baicalin and wogonoside using pH-peak-focusing counter-current chromatography using a polar solvent system containing trifluoroacetic acid. By using an n-butanol-ethyl acetate-methanol-water (1:4:0.5:5) two-phase solvent system, 493.2 mg of baicalin with a purity of 98.60% and 88.6 mg of wogonoside with a purity of 98.90% were obtained from 1.0 g of crude SR. The addition of polar solvents like trifluoroacetic acid in non-polar solvents effectively enhanced baicalin and wogonoside separation. Fan et al. (2021) developed an environmentally friendly method for grafting silica onto ionic liquids using ionic liquid 1-butyl-3-methylimidazolium bis (trifluoromethylsulfonyl) imide salt ([C4mim] NTf2) and ethanol as the reaction media. This method employed ionic liquid 1-propyl-3-methylimidazolium chloride ([C3mim] Cl) grafted silica ([C3mim] + Cl- @ SiO2) to adsorb and purify baicalin from SR root extract. The method successfully yielded baicalin with a purity of 96.50%.

In summary, chromatography methods yield the highest purity of flavonoids, with counter-current chromatography achieving purities exceeding 95%, surpassing the resin adsorption method. The ultrafiltration method provides flavonoids with a purity above 85%, comparable to resin adsorption. The acid precipitation method, however, results in the lowest purity, with a maximum of only 80.32%. High-speed counter-current chromatography offers the highest purity and extraction efficiency, reducing time, but requires complex operation and expensive equipment. Ultrafiltration, while energy-efficient and offering a high separation rate, suffers from significant membrane fouling and rapid flux decline, limiting the full recovery of active ingredients during large-scale production. The acid precipitation method, commonly used due to flavonoids’ multiple phenolic hydroxyl groups and weak acidity, produces lower purity compared to ultrafiltration and chromatography, and its use of acid introduces environmental and safety concerns. In contrast, the resin adsorption method is straightforward, cost-effective, and allows for resin regeneration and reuse. This method offers advantages such as ease of operation, high adsorption capacity, low cost, environmental sustainability, and a short production cycle, making it ideal for large-scale production and increasingly favored in recent years (Table 3).

**TABLE 3:** Purification methods of flavonoids.

Purification method	Purification conditions			Purity	References
Acid precipitation method	Raw material for extraction: SR medicinal herb; pH value: 1.0–2.0; holding temperature: 80 °C; holding time: 1 h; standing time: 24 h.			Baicalin 72.77%	Chen (2018)
	Raw material for extraction: SR root; pH value: 1.0–1.5; holding temperature: 80 °C; holding time: 2 h; standing time: 1 h.			Baicalin 80.32%	Zhang (2012)
	Raw material for extraction: dried root of SR; pH value: 1.0–2.0; holding temperature: 80 °C; holding time: 1 h; standing time: 12 h.			Baicalin 67.22%	Li (2011)
Ultrafiltration method	Raw material for extraction: SR decoction pieces; first 100K ultrafiltration column ultrafiltration and then 5K ultrafiltration column ultrafiltration; re-acid precipitation (PH value: 1.5–2.0; holding temperature: 80 °C; holding time: 30 min; standing time: 12–16 h).			Baicalin 89.98%	Wang et al. (2007)
	Raw material for extraction: dried root of SR; first 100K ultrafiltration column ultrafiltration; re-acid precipitation (PH value: 1.0–2.0; holding temperature: 80 °C; holding time: 1 h; standing time: 12 h).			Baicalin 91.47%	Li (2011)
Chromatography method	Resin adsorption method	HPD300 resin	Raw material for extraction: SR decoction pieces; the content of sample solution: 100 mg/mL; sample volume: 3 BV; washing volume: 7 BV; elution with 70% ethanol aqueous solution; elution volume 5 BV	Total flavonoids 88.15%.	Li and Liu (2017)
		Non-polar resin HPD-100	Raw material for extraction: dried root of SR; equilibrium time: 180 min; pH value: 5; sample volume: 2BV/ h; adsorption temperature: 25 °C; elution with 20%–40% ethanol aqueous solution.	Baicalin 85.70% Wogonoside 65.60%	Du et al. (2012)
		D104 macroporous resin	Raw material for extraction: dried root of SR; adsorption times: 3 times; standing time: 14 h; elution with 70% ethanol.	Baicalin 93.42%	Li (2011)
	Counter-current chromatography method	High-speed counter-current chromatography	Raw material for extraction: SR root; solvent system: ethyl acetate-methanol-1% acetic acid water (5:0.5:5).	Baicalin 99.20% Wogonoside 99.00%	Wu et al. (2005)
		PH-zone counter-current chromatography coupled with conventional counter-current chromatography	Raw material for extraction: dried root of SR; the solvent system of Ph-zone counter-current chromatography: n-butanol-ethyl acetate-water (2:3:5), trifluoroacetic acid was added to the organic phase to the final concentration of 10 mmol L ^−^ ^1^, and ammonia was added to the aqueous phase to the final concentration of 10 mmol L ^−^ ^1^. The solvent system of conventional counter-current chromatography: ethyl acetate-ethanol-3mmol L ^−^ ^1^ hydrochloric acid (10:1:10).	Baicalin 98.20% Wogonoside 98.90%	Wang R. G. et al. (2019)
		PH-peak-focusing counter-current chromatography	Raw material for extraction: dried root of SR; solvent system: n-butanol-ethyl acetate-methanol-water (1:4:0.5: 5).	Baicalin 98.60% Wogonoside 98.90%	Wang M. J. et al. (2020)
		Ionic liquid 1-propyl-3-methylimidazolium chloride ([C3mim] Cl) grafted silica ([C3mim] + Cl- @ SiO2)	Raw material for extraction: SR root; adsorption time: 10 min; pH value: 5; temperature: 25 °C	Baicalin 96.50%	Fan et al. (2021)

## 6 Metabolism *in vivo*

Metabolism refers to the process by which a drug’s chemical structure is altered by various drug-metabolizing enzymes within the body. Through metabolism, drugs can produce pharmacologically active metabolites that enhance their therapeutic effects, or generate toxic metabolites that may harm the body (Gu et al., 2022). Drug metabolism primarily occurs in the liver, kidneys, lungs, gastrointestinal tract, and skin, with the liver being the principal site of metabolic activity. As a key mechanism for eliminating drugs from the body, studying metabolism is essential for understanding the pharmacodynamic mechanisms of drugs *in vivo*.

### 6.1 Metabolic process *in vivo*

**FIGURE 2:**
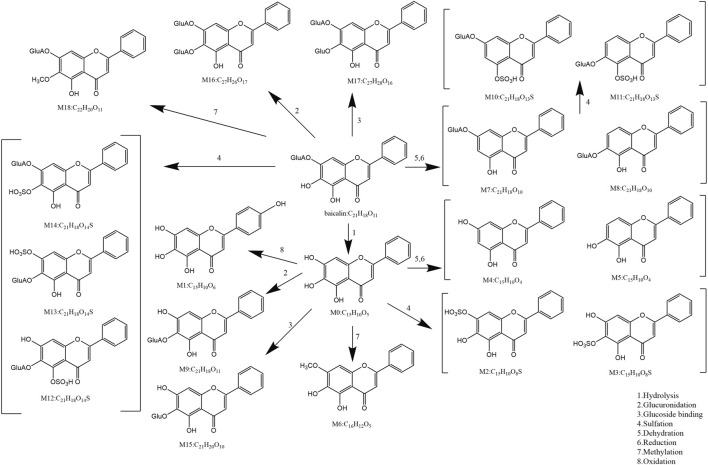
Possible metabolites of flavonoids.

The metabolism of flavonoids primarily involves phase I and phase II metabolic processes, including oxidation, reduction, and hydrolysis in phase I, as well as conjugation reactions such as glucuronidation, methylation, and sulfation in phase II (He et al., 2024). These processes are catalyzed by specific enzymes such as the cytochrome P-450 enzyme system (CYP-450) and transferases (Figure 2).

#### 6.1.1 Phase I metabolism: hydrolysis and oxidation

Initially, baicalin is catalyzed by intracellular β-glucuronidase, which removes the glucuronic acid group at the C7 position to form baicalein (Figure 2 M0). This step is critical for its metabolic activation, as baicalein, being more lipid-soluble than baicalin, more readily crosses cell membranes and exerts its effects on intracellular targets. The anti-inflammatory and antioxidant activities of baicalein are generally stronger than those of baicalin (Noh et al., 2016). In liver and intestinal epithelial cells, baicalein undergoes further metabolism by CYP450 enzymes, resulting in hydroxylation or demethylation reactions that produce metabolites such as 8-hydroxybaicalein and 4′-hydroxybaicalein (Figure 2 M1) (Xie et al., 2020). Additionally, baicalein is oxidized by tyrosinase and other quinone compounds like 4-methyl-o-benzoquinone and 4-tert-butyl-o-benzoquinone (Gąsowska-Bajger and Wojtasek, 2023). Liang et al. (2024) identified hydrolysis, hydroxylation, and methylation products of baicalin in rat serum, feces, and urine using ultra performance liquid chromatography-quadrupole/electrostatic field orbitrap high resolution mass spectrometry (UPLC-Q-Exactive Orbitrap-MS).

#### 6.1.2 Phase II metabolism: binding reaction

Baicalin and its metabolites, including baicalein, can also undergo glucuronidation catalyzed by uridine diphosphate-glucuronosyltransferases (UGTs), primarily at the C6 or C7 positions, resulting in mono- or di-glucuronide conjugates (Figure 2 M9, 16). This enhances water solubility, facilitating renal excretion, and is a major metabolic pathway in the liver. Sulfotransferase catalyzes the sulfation of hydroxyl groups in baicalin and its metabolites, producing water-soluble sulfated derivatives (Figure 2 M2, 3, 10-14), which are readily excreted *via* bile or urine (Fong et al., 2012). Furthermore, catechol-O-methyltransferase can methylate the phenolic hydroxyl group of baicalein to generate methoxy derivatives, such as 7-methoxybaicalein (Figure 2 M6), which can change its lipophilicity and blood-brain barrier penetration ability, thereby impacting its anti-inflammatory activity (Zhang et al., 2017). In a study, ultra-high performance liquid chromatography-tandem quadrupole time-of-flight mass spectrometry (UPLC-QTOF-MS) was used to identify 27 baicalin metabolites in rat urine, bile, and plasma, revealing that the main biotransformation pathways include methylation, sulfate conjugation, glucuronidation, isomerization, and hydroxylation (Yu et al., 2018).

#### 6.1.3 Metabolism of intestinal flora

In addition to phase I and phase II metabolism, baicalin is also influenced by the metabolism of intestinal flora *in vivo*. As a prodrug, baicalin requires hydrolysis by β-glucuronidase in the intestinal microbiota to form baicalein, which can then be effectively absorbed by intestinal epithelial cells. Lai et al. (2003) studied and compared the metabolic pharmacokinetics of baicalin and baicalein in rats following oral administration of equal molar doses of each compound. The results indicated that the absorption rate of baicalin was slower than that of baicalein. Baicalin must first be hydrolyzed by intestinal bacteria before absorption, while baicalein can be directly absorbed through the small intestine. However, due to the glucuronidation catalyzed by UGTs during phase II metabolism, baicalin can be reformed in the liver and enter the bloodstream, even if it is absorbed in the form of baicalein aglycone by intestinal microorganisms (Noh et al., 2016). A study using UPLC-QTOF/MS to analyze the metabolic process of baicalin in rats revealed that the pharmacokinetics, metabolism, and pharmacological effects of baicalin are largely dependent on the state of the intestinal microbiota. It was suggested that baicalin and antibiotics should not be used concurrently in clinical settings (Xing et al., 2017).

**FIGURE 3:**
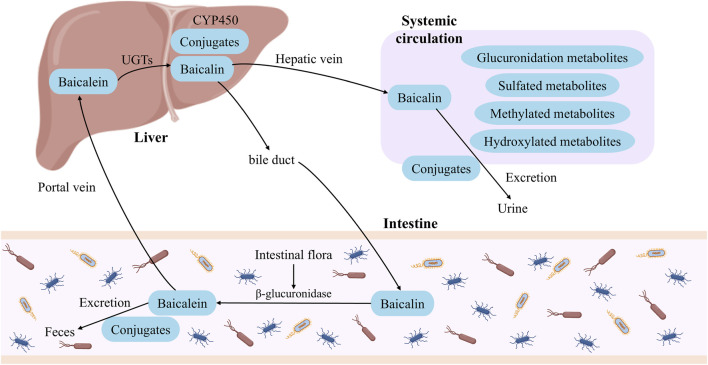
The metabolic process diagram of flavonoids *in vivo*.

In conclusion, the metabolism of flavonoids predominantly occurs in liver and intestinal epithelial cells (Figure 3). The liver serves as the primary metabolic organ, where UGTs and CYP-450 enzymes primarily mediate glucuronidation and oxidation reactions. Intestinal epithelial cells regulate the dynamic balance between baicalin and baicalein through the action of β-glucuronidase and UGTs. In the human body, baicalin is rapidly metabolized by intestinal flora into baicalein, which is a key step in baicalin absorption. Subsequently, baicalein, as the active form, undergoes complex *in vivo* metabolic processes and exerts a broad range of pharmacological effects.

### 6.2 Effect on pharmacological activity

The metabolites of flavonoids are closely linked to its pharmacological activities such as antiviral, anticancer, anti-inflammatory, antioxidant, and liver protective effects. Variations in metabolic pathways can lead to enhanced, diminished, or even novel pharmacological effects.

Zand et al. (2021) found that both baicalin and baicalein could inhibit the activity of SARS-CoV-2. At a concentration of 20μM, the inhibition rates of baicalein and baicalin on SARS-CoV-2 RNA-dependent RNA polymerase (RdRp) were 99.8% and 98%, respectively, indicating that baicalein exhibited a stronger antiviral effect. The study further revealed that baicalein specifically binds to SARS-CoV-2 RdRp, suggesting its potential for further research in COVID-19 treatment.

Bernasinska-Slomczewska et al. (2024) investigated the effects of baicalein and baicalin on breast cancer cells (MCF-7) and human endothelial cells (HUVEC-ST). The results showed that both compounds exhibited cytotoxic effects on these cell lines, with baicalein showing a stronger effect. According to the cell survival curves derived from the MTT method, the IC_10_ and IC_50_ values of baicalin were 36 μmol/L and 167 μmol/L, respectively, while the IC_10_ and IC_50_ values for baicalein were 9 μmol/L and 95 μmol/L, respectively. These findings suggest that both flavonoids can damage breast cancer cells, with baicalein being more potent. Both flavonoids induced PARP cleavage, exhibited pro-apoptotic activity, and caused DNA damage, contributing to their anticancer effects.

Lee et al. (2015) demonstrated that both baicalin and baicalein possess anti-inflammatory properties. They inhibit LPS-induced high permeability and leukocyte migration *in vivo* and reduce the production of tumor necrosis factor-α (TNF-α) and interleukin (IL)-6, as well as the activation of nuclear factor kappa-B (NF-κB) and extracellular regulated protein kinase (Erk) 1/2, which are beneficial for treating vascular inflammatory diseases. Experimental data indicated that baicalein exhibited stronger anti-inflammatory activity than baicalin. Liu et al. (2015) replaced baicalin in Sanhuang granules with baicalein to prepare Sudai Sanhuang granules and compared their antipyretic, analgesic, and anti-inflammatory effects using various animal models. The results showed that Sudai Sanhuang granules significantly inhibited the body temperature increase in dry yeast-induced fever in rats. Additionally, Sudai Sanhuang granules demonstrated a notable inhibitory effect on the pain response induced by acetic acid in mice, and the inhibition rate was 62.68%, surpassing that of the Sanhuang granules group. The swelling of rat toes induced by fresh egg white in the Sudai Sanhuang Granules group was significantly lower than that in the Sanhuang Granules group at 2, 3, and 4 h, indicating that baicalein had a stronger anti-inflammatory effect than baicalin. Sudai Sanhuang Granules exhibited superior antipyretic, analgesic, and anti-inflammatory effects, suggesting that replacing baicalin with baicalein could be a promising approach. Wang (2004) used a screening model for NO production by activated macrophages to explore the effect of baicalin metabolites on inflammation. The results demonstrated that both baicalein and its glucuronide metabolite, baicalein-6,7-diglucuronide, possess anti-inflammatory effects. However, when baicalin undergoes methylation to form 7-methoxybaicalein, its anti-inflammatory activity is significantly diminished. This finding, combined with studies on baicalin metabolism in rats, shows that glucuronidation is the predominant metabolic pathway, highlighting baicalin as a highly effective anti-inflammatory drug.

Wang (2004) also examined the inhibitory effect of baicalin and its metabolites on lipid peroxidation, specifically the production of malondialdehyde (MDA), a marker of oxidative stress. The results revealed that baicalin and its metabolites, baicalein and baicalein-6,7-diglucuronide, exhibited significant anti-lipid peroxidation effects in rat liver. Among them, baicalein demonstrated the highest antioxidant activity, followed by baicalin and baicalein-6,7-diglucuronide. After oral administration, baicalin is metabolized in the gastrointestinal tract into a more active hydrolytic metabolite, which not only facilitates drug absorption but also enhances baicalin’s therapeutic effects.

Shi et al. (2018) found that baicalein and baicalin induced nuclear factor erythroid 2-related factor 2 (Nrf2) phosphorylation by preventing its binding to kelch-like ECH-associated protein-1 (Keap1), thereby activating Nrf2 and alleviating acetaminophen (APAP)-induced hepatotoxicity. ERK 1/2 and protein kinase C (PKC) were identified as key regulators of Nrf2 phosphorylation induced by baicalein or baicalin. Importantly, the study showed that the hepatoprotective effect of baicalin in APAP-induced hepatotoxicity does not require conversion to its aglycone form, baicalein.

In conclusion, current research on the pharmacological effects of baicalin metabolites primarily focuses on its hydrolyzed form, baicalein, while there is relatively limited exploration of its glucuronidation and methylation products. Notably, the pharmacological activities of baicalin’s oxidation products, sulfated derivatives, and other metabolic forms remain underreported. Future studies should aim to elucidate the regulatory mechanisms of these metabolites on pharmacological activity, providing a comprehensive understanding of how flavonoids’ metabolic transformation correlates with its biological effects.

## 7 Immunological and pharmacological activities

The *in vivo* metabolic process of flavonoids in SR not only affects the bioavailability and tissue distribution of compounds, but also directly regulates its final biological effects. These aglycones, conjugates and their intestinal microbial metabolites formed by phase I and phase II metabolic transformation often have different activity characteristics and action targets from the prototype compounds. They are distributed throughout the body through blood circulation, and then interact with immune cells, inflammatory signaling pathways and various pharmacological targets. Therefore, understanding the relationship between the dynamic changes of metabolites and biological activities has become a key bridge to reveal the transformation of flavonoids in SR from ‘chemical components’ to ‘active molecules *in vivo*’. Based on this, the following will systematically elaborate the specific manifestations and molecular mechanisms of flavonoids and their metabolites in SR in terms of immunological regulation and pharmacological effects.

### 7.1 Immunomodulatory and antiviral effects

Flavonoids exhibit significant antiviral activity against respiratory syncytial virus, influenza virus, hepatitis virus, and the novel coronavirus. It demonstrates certain advantages whether used alone, in traditional Chinese medicine formulations, or in combination with other treatments.

Interferons (IFNs) are key cytokines in the antiviral innate immune response, and baicalin has been shown to increase the expression of type I and III IFNs, as well as their receptors (Li L. et al., 2023), playing a pivotal role in viral infections. Qin et al. (2022) found that oral administration of 200 mg/kg baicalin could promote the expression of ribosomal protein L13a and phosphorylated L13a in mouse models of respiratory syncytial virus infection, with ribavirin as a positive control drug. This resulted in a reduction in the expression of viral nonstructural protein (NS) 1 and matrix protein (M), inhibition of viral NS1 and NS2 protein transcription, and induction of type I IFN, IFNα, and IFNβ expression, thereby initiating an effective anti-respiratory syncytial virus innate immune response. There was no significant difference between the antiviral effect and the positive control drug ribavirin, indicating that baicalin has a significant antiviral effect.

Group B coxsackievirus (CVB) is one of the main viruses that cause viral myocarditis. Baicalein can block viral multiprotein processing and inhibit caspase-1 activation by inhibiting the activity of viral protease 2A (2A^pro^) of CVB3 to reduce the production of inflammatory factors. *In vitro*, baicalein can act on the early stage of CVB3 infection, reduce the levels of viral RNA, viral protein 3D and viral particles, inhibit CVB3 replication in myocardium, reduce myocardial injury and inflammatory response *in vivo*, thereby alleviating CVB3-induced myocarditis and improving the survival rate of infected mice. Baicalein may also have inhibitory effects on other enteroviruses such as coxsackievirus A16 (Dong et al., 2024). Zhang et al. (2023) studied the therapeutic effect of wogonin on human immunodeficiency virus type 1 (HIV-1) for the first time. The results showed that wogonin had a stronger inhibitory effect on HIV-1 than the known latent enhancer triptolide, and it also had the characteristics of low toxicity and long-term inhibition. The mechanism of wogonin was to specifically inhibit the expression of histone acetyltransferase p300, reduce the crotonylation level of histone H3 and H4 in the promoter region of HIV-1, and then realize the epigenetic silencing of HIV-1, inhibit viral transcription, and provide a potential new strategy for functional cure of HIV-1.

Additionally, baicalin has been shown to inhibit Hepatitis B virus (HBV) transcription and replication by enhancing the interaction between estrogen receptor (ER) α/β and hepatocyte nuclear factor (HNF)4α, which disrupts the transcriptional activation of HNF4α, leading to the downregulation of HNF1α transcription and expression, which is different from the mechanism of positive control drug entecavir. However, baicalin can be used in combination with entecavir to significantly enhance the inhibitory effect of entecavir on drug-resistant strains and solve the treatment problems caused by drug resistance (Xia et al., 2020). Some studies have taken HBV transfected cell lines and mouse acute HBV infection models as the research objects. By setting blank, solvent and positive control, after 48 h to 7 days of treatment, it was confirmed that baicalein inhibited HBV replication and antigen secretion through the coiled coil domain containing protein 88A (CCDC88A)-dependent autophagy pathway, and had no obvious toxicity. Its anti-HBV antigen effect is better than entecavir, which provides a new target and candidate drug for HBV treatment (Niu et al., 2025). Currently, a baicalin capsule is available as a listed drug for the treatment of chronic hepatitis B (CHB). Researchers have compared the efficacy of baicalin capsules before and after treatment. Yang et al. (2021) divided 79 patients with newly diagnosed CHB into two groups: a control group (23 patients, treated only with entecavir (ETV)) and a baicalin capsule group (56 patients, treated with ETV combined with baicalin capsules). After 4 weeks of treatment, 8.7% of patients in the control group (2/23) had a decrease in pregenomic ribonucleic acid (pgRNA) ≥1 × lg copy/mL, compared to 28.1% in the baicalin capsule group (16/56). Follow-up after 12 weeks showed that 54.0% of patients in the baicalin capsule group had a pgRNA decrease of ≥1 × lg copy/mL, significantly higher than the 26.3% in the control group. This study indicates that combining baicalin capsules with ETV treatment significantly reduces HBV pgRNA levels in patients with CHB, maintains immune balance, and improves the patients’ symptoms, suggesting its potential for widespread clinical application.

Flavonoids play a pivotal role in combating the global spread of the novel coronavirus. Wan et al. (2024) demonstrated that the combined treatment of baicalin and andrographolide reduced angiotensin-converting enzyme 2 (ACE2) levels, inhibited the binding of the SARS-CoV-2 spike S protein to the ACE2 receptor, and prevented viral entry into host cells. Additionally, this combination lowered pro-inflammatory cytokines such as IL-6 and TNF-α, mitigating the inflammatory response triggered by the virus, thus exerting an anti-coronavirus effect. Baicalin has also shown efficacy against other viruses, such as Marek’s disease virus (Yang F. et al., 2020), rotavirus (Song L. et al., 2021), and coxsackievirus (Wang M. J. et al., 2020).

**FIGURE 4:**
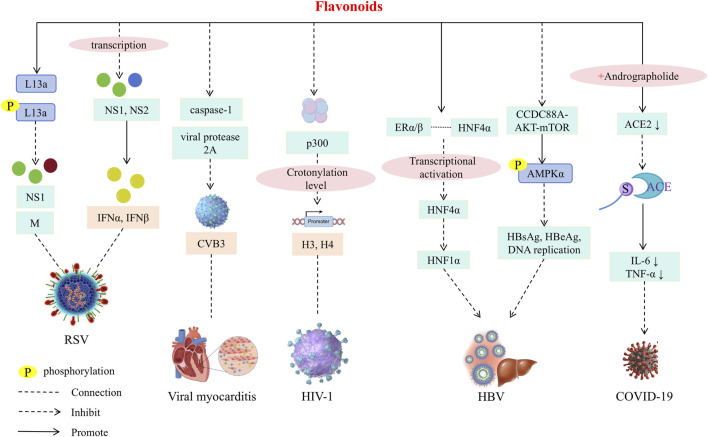
Antiviral mechanism of flavonoids.

The broad antiviral application of flavonoids is attributed to their ability to inhibit viral replication and transcription, block virus binding to receptors, suppress viral activity, regulate the expression of host cell functional proteins, and reduce inflammatory responses (Figure 4; Table 4).

**TABLE 4:** Antiviral effect of flavonoids.

Compound	Experimental subject	Experimental dose	Animal experimental drug delivery method	Minimal active concentration	Duration	Effects	References
Baicalin	The lung adenocarcinoma HEp-2 cell line Six-week-old male BALB/c mice (18–20 g) were infected with RSV intranasally	0.039,0.078,0.156,0.312 mg/mL 100, 200 mg/kg	Oral administration	*In vitro*: 0.0894 mg/mL *In vivo*: 100 mg/kg	*In vitro*: 72 h. *In vivo*: treatment was started 24 h after infection for 5 consecutive days.	Increase: Ribosomal Protein L13a, phosphorylated L13a, type I IFN, IFN-α, IFN-β Decrease: NS1, NS2, M	Qin et al. (2022)
Baicalein	HeLa and HEK293T cells Newborn Balb/c mice aged 7 days after birth were intraperitoneally inoculated with CVB3	6.25–400 μmol/L 10 mg/kg	Administered intraperitoneally	HeLa cells: 45.01 μmol/L HEK293T cells: 38.12 μmol/L	*In vitro*: 24 h. *In vivo*: treatment was started 24 h after infection for 5 consecutive days.	Decrease: caspase-1, viral protease 2A	Dong et al. (2024)
Wogonin	J-Lat 6.3, J-Lat 8.4, J-Lat 9.2, J-Lat 10.6, J-Lat 15.4, and J-Lat A2 cells	10–50 µM	None	40 µM	24 h	Decrease: p300, the crotonylation level of H3 and H4	Zhang et al. (2023)
Baicalin	pHBV1.2-transfected HepG2 cells Male BALB/c mouse (18–22 g) was hydrodynamically injected with 10 μg pHBV1.2 plasmid	0–400 µM 10, 20, 40 mg/kg	Oral administration	*In vitro*: 25 µM *In vivo*: 10 mg/kg	*In vitro*: 4 days *In vivo*: The second day after infection, the drug was administered for 7 consecutive days.	Increase: protein-protein interactions of ERα/β and HNF4α Decrease: HNF1α transcription activation	Xia et al. (2020)
Baicalein	HepG2, Huh7, and HepG2.215 cell lines BALB/c mouse was subjected to a hydrodynamic in jection of 10 μg of the pHBV1.2 plasmid	0–100 μM 20, 40, 80 mg/kg	Intraperitoneal injection	*In vitro*: 12.5 µM *In vivo*: 20 mg/kg	*In vitro*: 48 h to 6 days *In vivo*: 7 days	Increase: Phosphorylation of AMPKα Decrease: CCDC88A-AKT-mTOR, HBsAg, HBeAg, DNA replication	Niu et al. (2025)
Baicalin	Transgenic mice were infected with SARS-CoV-2 Cells infected with SARS-CoV-2	19.52 ± 4.77 μg/mL 10 mg/d	Gavage administration	*In vitro*: 25 μg/mL *In vivo*: 10 mg/d	*In vitro*: 24 h *In vivo*: 2 weeks	Decrease: ACE2, S proteins and ACE2 receptors binding, IL-6 and TNF-α	Wan et al. (2024)

At present, the potential of flavonoids in SR as a broad-spectrum antiviral immunomodulator has not been fully explored. Future research should focus on exploring the precise mechanism of its regulation of type I IFNs signaling pathway and mucosal immunity, especially the regulation of local immune network in the two major viral invasion portals of respiratory tract and intestine. We also developed a nano-delivery system based on the core structure of flavonoids in SR, targeting viral infection foci and immune organs to enhance their bioavailability and specificity. These studies will promote the transformation of flavonoids in SR from traditional antiviral Chinese medicine components to modern ' immune-antiviral ' synergistic treatment strategies.

### 7.2 Immunomodulatory and anti-inflammatory effects

Flavonoids have a strong anti-inflammatory effect, can inhibit the expression of inflammatory factors, such as TNF-α, IL, *etc.*, to reduce the inflammatory response.

In SR, flavonoids such as baicalin, oroxyloside, baicalein and scutellarein synergistically improve colitis through multiple targets and pathways. In a mouse model of colitis induced by dextran sulfate sodium salt, oral administration of baicalin solution (100 mg/kg) was shown to restore colon length and spleen index to near normal levels, alleviate ulcer severity, decrease the expression of CD14 and IL-6 in the colonic mucosa, reduce the binding of Toll-like receptor 4 (TLR4), and inhibit the activation of the myeloid differentiation primary response protein 88 (MyD88)/NF-κB p65 pathway, and the positive control drug mesalazine showed similar protective effects, can effectively improve the main indicators of the disease, thus effectively exerting an anti-colitis effect (Fu et al., 2021). Oroxyloside inhibits endoplasmic reticulum stress induced by DSS or LPS by activating peroxisome proliferator-activated receptor γ (PPARγ), downregulates the expression of endoplasmic reticulum stress-related proteins such as PERK, p-eIF2α, ATF4, and CHOP, and reduces the production of reactive oxygen species (ROS) and the secretion of pro-inflammatory factors such as IL-1β, IL-6, and TNF-α, and PPARγ antagonist GW9662 or PPARγ siRNA can reverse its protective effect (Tao et al., 2024). In DSS-induced mouse ulcerative colitis model, the therapeutic effect of oral administration of 20 mg/kg baicalein is equivalent to that of 200 mg/kg sulfasalazine. By regulating the ferroptosis pathway, baicalein can upregulate the expression of Nrf2, FTH1, GPX4, SLC7A11 and SLC3A2, downregulate the level of ACSL4, reduce the accumulation of Fe^2+^, MDA and ROS in colon tissue, and increase the activity of superoxide dismutase (SOD) and GSH to alleviate oxidative stress. It can also regulate the structure of intestinal flora, increase the abundance of beneficial bacteria, and reduce the level of pathogenic bacteria, thereby improving DSS-induced colitis in mice, indicating that baicalein has a significant anti-inflammatory effect, providing strong preclinical evidence for its potential as a therapeutic drug for ulcerative colitis (Lv et al., 2025). By inhibiting the phosphorylation and nuclear translocation of NF-κB, scutellarein, on the one hand, reduces the transcription and secretion of pro-inflammatory factors such as IL-6, IL-8 and TNF-α, on the other hand, upregulates the expression of tight junction proteins such as E-cadherin, Occludin and ZO-1 in colonic epithelial cells to maintain the integrity of the epithelial barrier. In the DSS mouse model and IL-1β-treated HT-29 intestinal epithelial cells, the therapeutic effect of 20 mg/kg dose was significantly better than that of 5-aminosalicylic acid (200 mg/kg), which provided a more potential candidate drug and experimental basis for the treatment of ulcerative colitis (Tang Q. et al., 2024). The above results indicate that flavonoids can be used for the treatment of colitis by regulating inflammatory signal activation, oxidative stress, endoplasmic reticulum stress, ferroptosis, dysbacteriosis, epithelial barrier damage, *etc.*

In a rat model of chronic cerebral hypoperfusion, baicalin promoted myelin regeneration and inhibited neuroinflammation by activating Wnt/β-catenin signaling and inhibiting NF-κB signaling, thereby improving cognitive impairment in rats with vascular dementia induced by chronic cerebral hypoperfusion (Xiao et al., 2023). Studies have taken cigarette smoke-induced cell inflammation model and mouse chronic obstructive pulmonary disease (COPD) model as the research objects. By setting blank, model, positive drug and pathway inhibitor control, it has been confirmed that baicalein can improve COPD pathological damage by inhibiting hypoxia-inducible factor 1-α (HIF1 α)-mediated oxidative stress and regulating CD8^+^ T cytotoxicity. In this study, high-dose baicalein (50 mg/kg) has the same effect as the standard anti-inflammatory drug dexamethasone (2 mg/kg) in improving lung function and lung pathological damage in COPD model mice, which provides a new choice for the treatment of COPD (Xiao et al., 2025). Scutellarin inhibits pyroptosis-related inflammation through a dual mechanism: on the one hand, it directly inhibits apoptosis-related spot-like protein oligomerization and blocks NLRP3, AIM2, NLRC4 inflammasome and non-classical pathway-mediated inflammasome activation; on the other hand, the pyroptosis executive protein Gasdermin D and its active fragment p30 are degraded by ubiquitin-dependent selective autophagy. Specifically, the E3 ubiquitin ligase TRIM21 catalyzes the K33-linked ubiquitination of the Lys51 site of p30/GSDMD, which is then recognized by the autophagy cargo receptor SQSTM1/p62 and mediates its degradation into the autophagy pathway, and ultimately inhibits the release of inflammatory factors (IL-1β, IL-18) caused by pyroptosis (Li et al., 2025).

The anti-inflammatory mechanism of flavonoids oroxylin A against different skin inflammation is clear and accurate. For LL-37-induced rosacea-like skin inflammation, oroxylin A plays a role by regulating the SIRT3-SOD2-NF-κB signaling pathway, activating SIRT3 to promote the deacetylation of SOD2 to enhance the scavenging ability of ROS and reduce ROS accumulation, thereby inhibiting NF-κB activation and nuclear translocation, reducing the secretion of pro-inflammatory factors and angiogenesis, and ultimately improving the characteristic erythema, telangiectasia and epidermal thickening of rosacea (Feng et al., 2024). For psoriasis-like skin inflammation, oroxylin A directly targets p62 (sequestosome 1), destroys the interaction between p62 and PKCζ by binding its PB1 domain, inhibits NF-κB activation, thereby inhibiting M1 macrophage polarization, and indirectly reducing Th17 cell differentiation and pro-inflammatory factor release, and ultimately reducing psoriasis-like skin lesions, epidermal hyperplasia, inflammatory cell infiltration and other injuries. In this study. At a dose of 80 mg/kg, oroxylin A showed a therapeutic effect comparable to that of cyclosporine A (30 mg/kg). It can significantly reduce skin inflammation, reduce immune cell infiltration, and reduce inflammatory factor levels, without obvious systemic side effects (Ma Y. et al., 2024).

**FIGURE 5:**
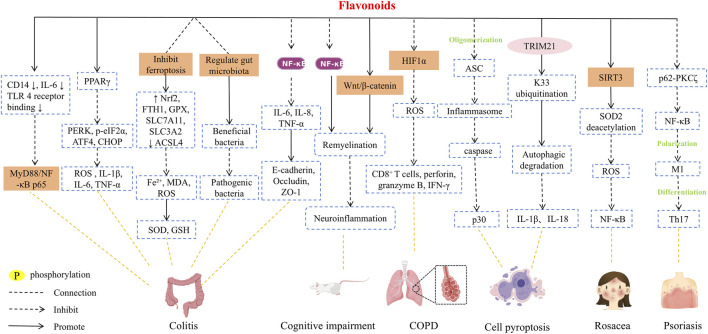
Anti-inflammatory mechanism of flavonoids.

The above studies have shown that flavonoids can be used to treat cognitive impairment, lung disease, skin inflammation and other diseases of vascular dementia induced by colitis and chronic cerebral hypoperfusion. Its anti-inflammatory effects are primarily mediated through mechanisms such as reducing inflammatory cell infiltration, inhibiting NF-κB nuclear translocation, modulating autophagy, regulating gene expression, and blocking key signaling pathways (Figure 5; Table 5).

**TABLE 5:** Anti-inflammatory effect of flavonoids.

Compound	Experimental subject	Experimental dose	Animal experimental drug delivery method	Minimal active concentration	Duration	Effects	References
Baicalin	RAW264.7 cells LPS-induced inflammatory BALB/c mouse model DSS-induced ulcerative colitis C57BL/6 mouse model	6.25–200 μM 100 mg/kg	Oral administration	*In vitro*: 12.5 µM *In vivo*: 100 mg/kg	*In vitro*: 24 h *In vivo*: 3–5 days	Decrease: TLR4/NF-κB p65pathway, CD14, MyD88	Fu et al. (2021)
Oroxyloside	RAW 264.7 mouse macrophage cells Female C57BL/6 mice were administered drinking water containing 4% (W/V) DSS (model)	100 μM 80 mg/kg	Oral administration	*In vitro*: 100 µM *In vivo*: 80 mg/kg	*In vitro*: 24 h *In vivo*: 10 days	Increase: PPARγ Decrease: PERK, p-eIF2α, ATF4, CHOP, ROS, IL-1β, IL-6, TNF-α	Tao et al. (2024)
Baicalein	C57BL/6J mice was initiated by 4% (w/v) DSS solution	10, 20 mg/kg	Gavage administration	10 mg/kg	7 days	Increase: Nrf2, FTH1, GPX4, SLC7A11, SLC3A2, SOD, GSH Decrease: ACSL4, Fe^2+^, MDA, ROS	Lv et al. (2025)
Scutellarein	Human intestinal epithelial cell HT-29 Male C57BL/6 mice were challenged with a 3% concentration of DSS	1–30 μM 5, 10, 20 mg/kg	Intragastric gavage	*In vitro*: 10 µM *In vivo*: 10 mg/kg	*In vitro*: 24 h *In vivo*: 7 days	Increase: E-cadherin, Occludin, ZO-1 Decrease: NF-κB, IL-6, IL-8, TNF-α	Tang et al. (2024a)
Baicalin	BCCAO CCH rat model	50, 100 mg/kg	Gavage administration	100 mg/kg	28days	Increase: MBP, Olig2, GSK3β, β-catenin Decrease: NF-κB	Xiao et al. (2023)
Baicalein	The BEAS-2B human bronchial ECs COPD inflammation model was established by passive inhalation of cigarette smoke in C57BL/6J mice	10–80 μM 10, 50 mg/kg	Intragastric gavage	*In vitro*: 20 µM *In vivo*: 10 mg/kg	*In vitro*: 24 h *In vivo*: 4 weeks	Decrease: HIF1α, ROS, CD8^+^ T cells, perforin, granzyme B, IFN-γ	Xiao et al. (2025)
Scutellarin	Human embryonic kidney cells HEK-293T, ATG5-KO HEK-293T, and immortalized bone marrow-derived macrophages iBMDMs Human myeloid leukemia mononuclear cells THP-1 and PBMCs C57BL/6 J mice	50–200 μM 10 mg/kg	Intraperitoneal injection	*In vitro*: 100 µM *In vivo*: 10 mg/kg	*In vitro*: 24 h *In vivo*: 6 h	Increase: K33 ubiquitination, autophagic degradation Decrease: ASC oligomerization, caspase, p30, IL-1β、IL-18	Li et al. (2025)
Oroxylin A	HaCaT cells LL-37-induced mouse model	10 μM 20 mg/kg	Intraperitoneal injection	*In vitro*: 10 µM *In vivo*: 20 mg/kg	*In vitro*: 12 h *In vivo*: 2 days	Increase: SIRT3 Decrease: SOD2 deacetylation, ROS, NF-κB	Feng et al. (2024)
Oroxylin A	RAW264.7, THP-1, HEK293T Imiquimod-induced psoriasiform mice model	2–50 μM 20, 40, 80 mg/kg	Intragastric administration	*In vitro*: 2 µM *In vivo*: 20 mg/kg	*In vitro*: 24 h *In vivo*: 14 days	Decrease: p62-PKCζ binding, NF-Κb, M1 polarization, Th17	Ma et al. (2024a)

Most inflammatory diseases have local lesions (such as intestinal inflammation, skin inflammation, *etc.*), and systemic administration can easily lead to systemic immunosuppression. In the future, it is necessary to develop targeted local drug delivery preparations, such as intestinal targeted sustained-release capsules and skin external gel, so as to achieve accurate release of drugs at the lesion site, reduce the impact on the systemic immune system, and improve the specificity of treatment.

### 7.3 Immunomodulatory and antibacterial effects

Flavonoids, like other bactericidal agents such as penicillins and cephalosporins, exhibits inhibitory and bactericidal effects against a variety of bacteria, making it useful for treating a range of infectious diseases. As the use of antibiotics has increased, so has the rise of drug resistance in pathogens, highlighting the importance of screening antibacterial active ingredients from traditional Chinese medicine.

Flavonoids exert antibacterial effects against different pathogenic bacteria through precise mechanisms. The antibacterial effect of baicalin can be categorized into direct and indirect effects. Baicalin regulates mitochondrial homeostasis in macrophages, enhancing their antibacterial activity and providing protective effects in chronic infectious diseases such as pneumonia caused by *Staphylococcus aureus* (*S. aureus*) (Zhao et al., 2022). In a study, the minimum inhibitory concentration (MIC) of tea polyphenols against methicillin-resistant *S. aureus* was determined to be 760 μg/mL, which served as the control. At a concentration of 5 MIC, baicalin significantly inhibited bacterial growth. Crystal violet staining revealed that baicalin could also inhibit biofilm formation. Real-time polymerase chain reaction (PCR) results indicated that baicalin reduced the mRNA levels of virulence-related factors hla and argA in *S. aureus*. This demonstrated baicalin’s potential for treating *S. aureus* infections (Zhang S. et al., 2020). In this study, *Aeromonas hydrophila* and grass carp infection model were used as the research object. By setting blank, solvent, model and positive drug control, after 2–72 h *in vitro* intervention and 4 h *in vivo* observation, it was confirmed that baicalein showed significant antibacterial activity against *A. hydrophila in vitro* and *in vivo*. Baicalein can destroy bacterial membrane permeability, inhibit biofilm formation and bacterial motility *in vitro*. The MIC was 40 μg/mL and the minimum bactericidal concentration (MBC) was 80 μg/mL. *In vivo*, it can reduce bacterial load, inhibit pro-inflammatory factors IL-1β, IL-8, *etc.*, and increase the expression of antioxidant genes CAT and SOD to regulate immunity (Qiu et al., 2025). Scutellarein inhibits the activity of *Acinetobacter baumannii* by directly targeting polyphosphate kinase 1 (PPK1), thereby inhibiting bacterial motility, biofilm formation and retention. *In vivo*, it can reduce the bacterial load and pathological damage of the infection model and alleviate inflammation. Both scutellarein (20 mg/kg) and the positive control drug meropenem (5 mg/kg) significantly reduced the colonization of *A. baumannii* in lung and liver tissues of mice, and alleviated the organ damage caused by infection (Song et al., 2024).

Baicalin can also be combined with antibiotics to enhance the antibacterial effect. When combined with azithromycin, baicalin significantly inhibits biofilm detachment, upregulates the relative transcription of genes related to the WalK/R system, and reduces the number of inflammatory cells. This combination reduces the MIC of azithromycin in biofilm by 4–512 times, enhancing its bactericidal efficacy against multidrug-resistant *Staphylococcus saprophyticus* biofilm (Wang J. et al., 2022).

**FIGURE 6:**
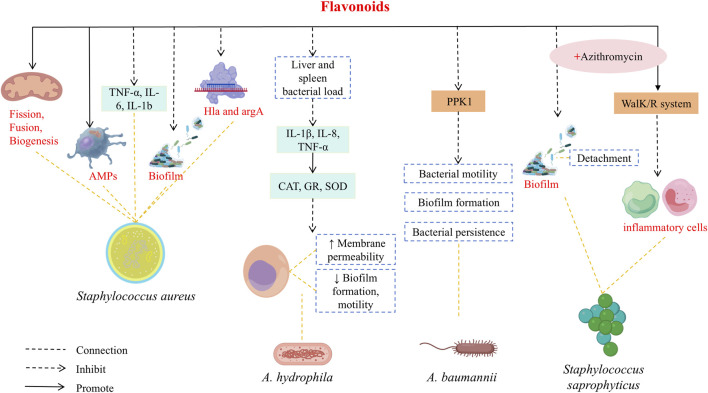
Antibacterial mechanism of flavonoids.

In summary, flavonoids’ antibacterial mechanism primarily enhances macrophage antibacterial activity, prevents bacterial biofilm formation, and suppresses bacterial gene expression. Moreover, flavonoids can also work synergistically with antibiotics to accelerate biofilm destruction, facilitate antibiotic penetration, increase bactericidal efficacy, and reduce biofilm resistance, thereby enhancing overall antibacterial effects (Figure 6; Table 6).

**TABLE 6:** Antibacterial effect of flavonoids.

Compound	Experimental subject	Experimental dose	Animal experimental drug delivery method	Minimal active concentration	Duration	Effects	References
Baicalin	RAW264.7 macrophages *S. aureus* infection model in C57BL/6 mice	20, 40, 80 μM 50 mg/kg	Intraperitoneal injection	*In vitro*: 40 µM *In vivo*: 50 mg/kg	*In vitro*: 18 h *In vivo*: 72 h	Increase: AMPs (Reg3b and SA1008), The mRNA levels of Opa1, Mfn1, ERRa, and NQO1, The protein expression of Mfn1 and Nrf1, Phosphorylation of Drp1 Decrease: TNF-a, IL-6, IL-1b	Zhao et al. (2022)
Baicalin	*S. aureus* strain (SA002) BALB/c mice nasally infected with MRSA	380μg/mL-3.8 mg/mL 80 mg/kg	Intranasal administration	*In vitro*: 760 μg/mL *In vivo*: 80 mg/kg	*In vitro*: 24 h *In vivo*: 5 days	Decrease: The mRNA levels of hla and argA, Biofilm	Zhang S. et al. (2020)
Baicalein	*A. hydrophila* Grass carp infected by *A.hydrophila*	1.25–320 μg/mL 2.5, 5, 10 mg/kg	Intraperitoneal injection	*In vitro*: 40 μg/mL *In vivo*: 2.5 mg/kg	*In vitro*: 72 h *In vivo*: 4 h	Increase: CAT, GR, SOD Decrease: IL-1β, IL-8, TNF-α	Qiu et al. (2025)
Scutellarein	A549 (human NSCLC cell line), B2B (human bronchial epithelium cell line), and RAW (mouse macrophage cell line) cells A mouse model of pneumonia inoculated with *A. baumannii*	8–128 μg/mL 10, 20 mg/kg	Oral and subcutaneous administration	*In vitro*: 16 μg/mL *In vivo*: 10 mg/kg	*In vitro*: 48 h *In vivo*: 5 days	Decrease: PPK1, bacterial motility, biofilm formation	Song et al. (2024)
Baicalin	MDRSS Mouse cutaneous abscess infection model	562.5–9000 mg/L 30 mg/kg	Subcutaneous injection	*In vitro*: 562.5 mg/L *In vivo*: 30 mg/kg	*In vitro*: 24 h *In vivo*: 3 days	Increase: WalK/R system Decrease: Biofilm detachment, The number of inflammatory cells	Wang J. et al. (2022)

Excessive immune response can lead to tissue damage at the site of infection, while low immunity can easily lead to persistent bacterial infection. In the future, it is necessary to further study how flavonoids in SR balance the relationship between antibacterial and immune regulation, and to clarify the difference in regulation between the early stage of infection (enhancing immune clearance of bacteria) and the late stage of infection (inhibiting immune overreaction), so as to provide a basis for drug regimen in different stages of infection.

### 7.4 Immunomodulatory and antitumor effects

Flavonoids can inhibit the proliferation and metastasis of tumor cells and has a certain antitumor effect. For liver cancer and liver fibrosis-related liver cancer, it can exert anti-tumor effects by targeting specific pathways. Through the suppression of the ROCK1/glycogen synthase kinase-3 beta (GSK-3β)/β-catenin signaling pathway, baicalin inhibits the proliferation, metastasis, and invasion of hepatocellular carcinoma (HCC) cells, induces cell cycle arrest in the G0/G1 phase, and promotes apoptosis. Molecular docking studies have shown that baicalin effectively binds to ROCK1 protein, suggesting that ROCK1 may be one of the direct targets of baicalin in inhibiting HCC growth and metastasis (Sun et al., 2023). Wogonin activates the Hippo-YAP/TAZ pathway, promotes YAP/TAZ phosphorylation, downregulates downstream AXL and CCN1/CCN2, enhances the radiosensitivity of liver cancer and inhibits tumor growth (Xu et al., 2025). In a study using a mouse liver cancer xenograft model, baicalein was found to target ubiquitin-specific protease 21 (USP21), promoting the ubiquitination and degradation of HIF-1α. This core mechanism led to the downregulation of tumor-related proteins and regulation of the tumor immune microenvironment. Notably, baicalein demonstrated superior efficacy compared to the broad-spectrum deubiquitinating enzyme inhibitor PR-619, positioning it as a more promising candidate drug for USP21-targeted liver cancer therapy, particularly in terms of precisely inhibiting tumor progression and reshaping the immune microenvironment (Jia et al., 2025). Oroxyloside inhibits oxidative stress and IL-6-mediated inflammation through a PPARγ-dependent AMPK-ULK1 autophagy pathway, thereby blocking hepatic stellate cell activation. It exhibits dual anti-fibrotic and anti-hepatocellular carcinoma effects, with its efficacy dependent on hepatic Atg5 expression. Its anti-fibrotic efficacy is comparable to that of the positive control drug pioglitazone, establishing it as a novel therapeutic candidate and target for chronic liver disease management (Liu et al., 2025). Scutellarin covalently binds to the Cys297 site of isochlorate dehydrogenase 1 to promote the formation of its active dimer, increase the production of α-ketoglutarate to degrade HIF-1α, inhibit the glycolysis of liver cancer cells, activate the tumor immune microenvironment, increase the infiltration of CD4^+^/CD8^+^ T cells, downregulate PD-L1, and achieve anti-tumor effects (Cui et al., 2024).

Baicalin exerts antitumor activity by inducing ferroptosis of tumor cells. Baicalin physically interacts with Nrf2, a key regulator of ferroptosis, and induces its ubiquitin degradation, thereby destabilizing Nrf2. This results in the inhibition of downstream targets, such as glutathione peroxidase 4 (GPX4) and the X cystine/glutamate antiporter (Xct), which are involved in ferroptosis regulation. This mechanism stimulates ferroptosis, contributing to baicalin’s anti-osteosarcoma activity (Wen et al., 2023). While the full mechanism of baicalin against osteosarcoma remains to be fully elucidated, these findings highlight its potential as a therapeutic agent.

Oroxylin A can directly bind to the endoplasmic reticulum molecular chaperone protein GRP94, block its interaction with the E3 ubiquitin ligase MDM2, thereby inhibiting MDM2-mediated XPA protein ubiquitination and proteasome degradation pathway. XPA is the core protein of the nucleotide excision repair (NER) system. Its enhanced stability significantly improves the ability of DNA damage repair, accelerates the removal of UVB-induced photoproducts such as CPD and 6–4 PP, and reduces genomic instability. At the same time, oroxylin A indirectly inhibits the inflammatory response by regulating XPA-dependent NER function, reducing inflammatory cell infiltration and cytokine expression, and ultimately delaying the occurrence and development of skin tumors (Dou et al., 2025). Paclitaxel can inhibit the proliferation and migration of breast cancer stem cells (BCSCs). Taking paclitaxel as a positive control, the inhibitory effect of scutellarin on BCSCs can be compared. The results show that scutellarin plays an anti-tumor role by regulating the key signaling pathways of BCSCs through multiple targets. It can inhibit the Wnt/β-catenin pathway, reduce the expression of downstream stem cell-related proteins, and weaken the self-renewal ability of breast cancer stem cells, inhibition of NF-κB signaling pathway, reduce the expression of IKK-α, IκB-α and other proteins, thereby inhibiting inflammation-related proliferation and migration. At the same time, it regulates the PTEN/Akt/mTOR pathway, inhibits the phosphorylation of Akt and mTOR, hinders the process of epithelial-mesenchymal transition, and reduces the invasion and metastasis of BCSCs. In addition, Scutellarin can also significantly reduce the expression of BCSCs marker CD44, inhibit its sphere formation, tumor formation and migration ability, and effectively inhibit tumor growth and lung metastasis *in vitro* and *in vivo* (Ma H. et al., 2024). The anti-tumor effect of baicalin is further enhanced when combined with other drugs. Shehatta et al. (2022) reported that baicalin, in combination with 5-fluorouracil, significantly reduced serum levels of NF-ĸB, IL-1β, and VEGF in mice, leading to decreased inflammation and angiogenesis. Additionally, apoptotic genes such as caspase-3, inducible gene 3 (p53), and Bcl-2-associated X protein (Bax) were upregulated, while the anti-apoptotic gene Bcl-2 was downregulated. These findings suggest that baicalin enhances the sensitivity of breast cancer cells to 5-fluorouracil and effectively inhibits tumor growth.

**FIGURE 7:**
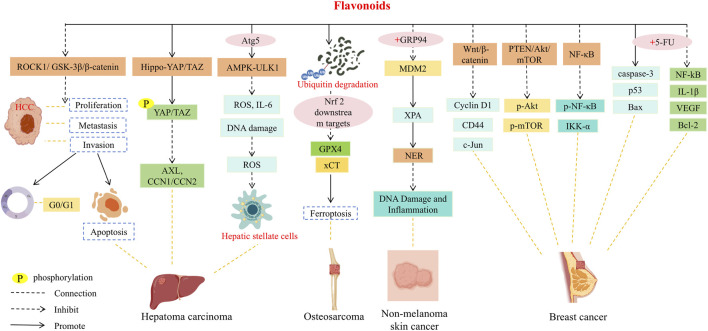
Anti-tumor mechanism of flavonoids.

Flavonoids have also shown inhibitory effects on other cancers, including prostate cancer (Yu et al., 2020), colorectal cancer (Song et al., 2022), lung cancer (Sui et al., 2021), and colon cancer (Dou et al., 2018). Its antitumor activity is characterized by its involvement in multiple pathways, targets, and mechanisms. Flavonoids can arrest the cell cycle, induce apoptosis, inhibit cell proliferation, modulate apoptotic genes, suppress signaling pathways, and hinder cell invasion and migration. Furthermore, flavonoids’ combination with other drugs enhances its anti-tumor efficacy (Figure 7; Table 7).

**TABLE 7:** Antitumor effect of flavonoids.

Compound	Experimental subject	Experimental dose	Animal experimental drug delivery method	Minimal active concentration	Duration	Effects	References
Baicalin	HCC (Hep3B and MHCC-97H) cells MHCC-97H xenograft tumor nude mouse model	10–120 μM 80 mg/kg	Gavage administration	*In vitro*: 40 µM *In vivo*: 80 mg/kg	*In vitro*: 48 h *In vivo*: 20 days	Increase: GSK-3β, p-β-catenin, Bax Decrease: HCC growth, ROCK1, p-GSK-3β, β-catenin, Bcl-2、C-myc、Cyclin D1、MMP-9、VEGFA	Sun et al. (2023)
Wogonin	SMMC-7721 and HCC-LM3 HCC cell lines HCC-LM3 cells were injected into nude mice	50, 100 μM 50, 100 mg/kg	Intraperitoneal injection	*In vitro*: 50 µM *In vivo*: 100 mg/kg	*In vitro*: 48 h *In vivo*: 30 days	Increase: Hippo-YAP/TAZ, Phosphorylation of YAP/TAZ Decrease: AXL, CCN1/CCN2	Xu et al. (2025)
Baicalein	Human HEK-293T cells, the human HCC cells (HepG2 and Bel7402) and mouse HCC cells (H22) Mouse hepatoma cell line H22 was subcutaneously inoculated into wild-type C57BL/6 mice.	10–80 μM 10, 20 mg/kg	Intraperitoneal administration	*In vitro*: 20 µM *In vivo*: 10 mg/kg	*In vitro*: 72 h *In vivo*: 2 weeks	Increase: T cell infiltration Decrease: USP21, HIF-1α, PD-L1, FoxM1	Jia et al. (2025)
Oroxyloside	Human hepatocytes L02 and hepatic stellate cells LX2 HCC model mice Liver-specific Atg5 knockout mouse model	50 μM 20, 40, 80 mg/kg	Intraperitoneal injection	*In vitro*: 50 µM *In vivo*: 20 mg/kg	*In vitro*: 24 h *In vivo*: 6 weeks	Increase: AMPK-ULK1 Decrease: ROS, IL-6	Liu et al. (2025)
Scutellarin	HepG2、Huh7、H22 cells BALB/c mice were subcutaneously transplanted with H22 hepatoma cells (IDH1 wild type or overexpression)	5–200 μM 60, 100 mg/kg	Intraperitoneal injection	*In vitro*: 5 µM *In vivo*: 60 mg/kg	*In vitro*: 48 h *In vivo*: 28 days	Increase: IDH1 dimer, α-KG, CD4^+^/CD8^+^ T cells Decrease: HIF-1α, PD-L1	Cui et al. (2024)
Baicalin	Human OS cell lines including MG63, 143B and human bone marrow mesenchymal stem cells Balb/C mice injected with MG63 cells	60–120 μg/mL 200 mg/kg	Intraperitoneal injection	*In vitro*: 60 µM *In vivo*: 200 mg/kg	*In vitro*: 7 days *In vivo*: 14 days	Increase: Ubiquitin degradation, ferroptosis Decrease: GPX4, xCT	Wen et al. (2023)
Oroxylin A	Human immortal keratinocyte line (HaCaT) cells UVB-irradiated SKH-1 hairless mouse model	12.5–400 μM 100 μmol/L	Gavage administration	*In vitro*: 50 µM *In vivo*: 100 μmol/L	*In vitro*: 24 h *In vivo*: 27 weeks	Increase: XPA, NER Decrease: MDM2, IL-1β, IL-6, F4/80 macrophages, MPO neutrophils	Dou et al. (2025)
Scutellarin	Breast cancer stem cells MDA-MB-231-induced BCSCs were seeded into mouse mammary fat pads	100μM, 200μM, 300 μM 50, 100 mg/kg	Intraperitoneal injection	*In vitro*: 100 µM *In vivo*: 100 mg/kg	*In vitro*: 48 h *In vivo*: 1 month	Decrease: Cyclin D1, CD44, c-Jun, p-Akt, p-mTOR, p-NF-κB, IKK-α, Ki67, CD44	Ma et al. (2024b)
Baicalin	Ehrlich solid tumor-mice model	15, 50 mg/kg	Intraperitoneal injection	50 mg/kg	21 days	Increase: caspase-3, p53, Bax Decrease: NF-kB, IL-1β, VEGF, Bcl-2	Shehatta et al. (2022)

The effect of a single component often cannot fully reflect the overall efficacy of traditional Chinese medicine compound. In the future, the synergistic compatibility law between flavonoids in SR and other components in SR, or with other classical anti-tumor active ingredients of traditional Chinese medicine should be further studied. By using the methods of system pharmacology and network pharmacology, the scientific connotation of its integrated regulation of tumor immune cycle was revealed from the perspective of multi-component-multi-target-multi-pathway.

In summary, flavonoids exhibit a wide range of pharmacological effects, including immunomodulatory and anti-inflammatory, antibacterial, antiviral, and antitumor properties. It plays a multi-target, integrated regulatory role through modulation of signaling pathways such as NF-κB, MAPK, and Nrf2. However, while its significant pharmacological activity is well-established, further systematic research on its toxicity profile and safe dosage range is needed to solidify its clinical application foundation.

## 8 Toxicity

While flavonoids exhibit multi-pathway, multi-target, and multi-link pharmacological effects, its potential toxicity risk has become increasingly evident as research progresses. Modern toxicological studies confirm that flavonoids’ toxic effects are multidimensional. High doses of flavonoids can cause significant liver and kidney damage, with *in vitro* experiments showing selective toxicity to specific cell types, such as senescent stem cells and embryonic cells. Immune-related studies suggest that flavonoids may trigger allergic reactions by directly activating immune cells or inducing antibody production.

For instance, studies have shown that administering 200 mg/kg of baicalin to silicosis model rats significantly increased serum aspartate aminotransferase, alkaline phosphatase, and urea nitrogen levels compared to the model group, leading to glomerular atrophy, renal tubular atrophy, and epithelial cell necrosis. These findings indicate that high-dose baicalin has notable liver and kidney toxicity (Zhang Y. Y. et al., 2021). Additionally, when baicalin concentrations exceed 50 μM/L, the survival rate of human bone marrow mesenchymal stem cells in elderly individuals significantly decreases, demonstrating substantial cytotoxicity (Zhai et al., 2020). Baicalin also inhibits the differentiation of embryonic stem cells (D3) and embryonic fibroblasts (BALB/c 3T3), suggesting weak embryotoxicity (Zhang et al., 2012). Moreover, baicalin can activate HEK293 cells expressing Mrgprb2, inducing pseudoallergic reactions both *in vitro* and *in vivo* (Wang J. et al., 2020). Studies have linked the anaphylactoid reactions of Shuanghuanglian injection to baicalin. After multiple immunizations, exogenous IgE antibodies were produced in rabbits, resulting in allergic reactions (Cao et al., 2024; Deng et al., 2017). Flavonoids such as baicalin and wogonin in Pudilan Oral Liquid can mediate the developmental toxicity of zebrafish embryos by interfering with cell cycle progression, destroying DNA replication, inhibiting steroid hormone biosynthesis and showing phytoestrogen-like activity (Chen et al., 2025). Studies have shown that wogonin has cytotoxicity and genotoxicity to RAW264.7 macrophages when the concentration exceeds 0.1 mM. By activating death receptors and mitochondrial pathways, it causes macrophage DNA damage, caspase cascade reaction and apoptosis in a concentration-dependent manner (Tsai et al., 2024). Additionally, the extract of SR root in cosmetics has been found to cause allergic contact dermatitis (Badaoui, 2022).

The studies above review the latest research on flavonoids’ hepatorenal toxicity, cytotoxicity, embryotoxicity, and immunogenicity risks, revealing that the toxicity of flavonoids is dose-dependent and mechanism-diverse. Notably, its toxicity risk intensifies in pathological models and special populations and is closely related to the clinical dosage forms commonly used. These findings not only provide an experimental basis for determining the safe dosage of flavonoids and identifying at-risk populations but also emphasize the need for future research to focus on the toxic mechanisms of flavonoids and strategies to mitigate these effects. Such efforts would facilitate its transition from a natural active ingredient to a safe and effective clinical drug.

## 9 New dosage forms

With the continuous advancement of traditional Chinese medicine modernization, enhancing the properties of active monomers through scientific methods has become a key area of research. Due to the poor lipid solubility or water solubility of some flavonoids, the oral bioavailability is low, which significantly limits its clinical efficacy. As a result, novel technologies are being employed to develop new formulations that improve its bioavailability, providing a foundation for its broader clinical application.

### 9.1 Nano formulations

**FIGURE 8:**
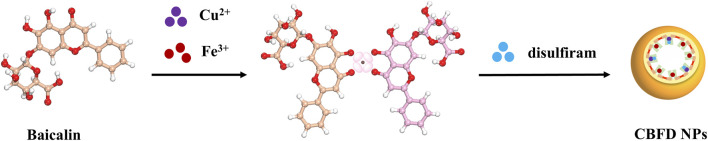
Cbfd NPs.

**FIGURE 9:**
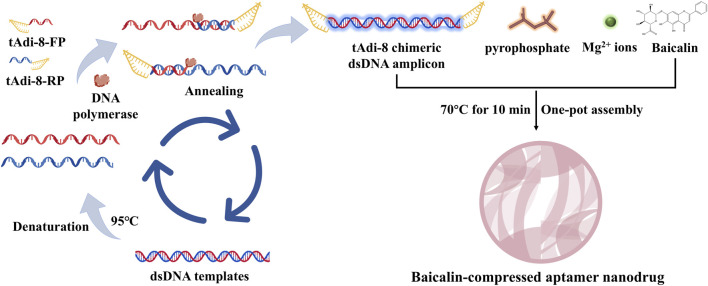
Baicalin-compressed-aptamer-nanodrug.

To enhance flavonoids’ bioavailability, various nano-formulations have been developed, including nanoparticles and nanocapsules. Fan et al. (2024) systematically investigated the coordination between baicalin and metal ions, such as Cu^2+^ and Fe^3+^, leading to the design and preparation of disulfiram co-loaded coordination nanoparticles, called CBFD NPs. These nanoparticles address the solubility issue of baicalin. Upon tracheal intubation, they efficiently accumulate in lung tissue, rapidly penetrate the lung mucosa, alleviate lung injury symptoms, and exhibit no significant toxicity or side effects. This formulation shows potential for targeted therapy in conditions such as one-lung ventilation-induced lung injury and reperfusion injury (Figure 8). Additionally, a one-pot assembly of baicalin-compressed-aptamer-nanodrug has been developed for synergistic targeted therapy of obesity (Figure 9). Utilizing PCR technology, a double-stranded DNA microflower structure was constructed, and the tailored Adipo-8 aptamer was incorporated to target adipocytes. Baicalin was embedded in the structure, serving a triple function: reducing lipid content, shrinking nanoparticle size from 2.7 μm to 214 nm, and activating thermogenic protein uncoupling protein 1 (UCP1). The nanodrug displayed high drug loading (with baicalin loading up to 86.21 g/g DNA), pH-responsive release (release in acidic environments over 72 h), and excellent biocompatibility. *In vitro* studies demonstrated that the baicalin-compressed-aptamer-nanodrug significantly enhanced adipocyte thermogenesis, resulting in a 2.5-fold increase in UCP1 expression, and reduced lipid droplet accumulation. *In vivo* experiments revealed that the nanodrug targeted visceral white fat, leading to a 28.75% reduction in body weight and a 21.43% decrease in triglyceride levels in obese mice. This method is cost-effective and allows for rapid preparation, with assembly completed in just 10 min, presenting a novel multifunctional nanodrug platform for obesity treatment (Tian et al., 2023). Polyethyleneimine-passivated heparin carbon dots, known for their exceptional cell penetration efficiency, localized entirely in the cytoplasm. Baicalin-loaded polyethyleneimine-passivated heparin carbon dots effectively arrested cells in the G2/M phase, exhibited enhanced apoptotic activity, and demonstrated stronger anticancer effects, indicating their potential as a promising nano-anticancer agent (Meher et al., 2023). Baicalein can coordinate with Fe^3+^ to form Fe@Ba metal-polyphenol self-assembled nanozymes with CAT and SOD-like activities, which can scavenge ROS, inhibit lipid peroxidation and ferroptosis, thereby alleviating cisplatin-induced acute kidney injury (AKI) (Xiong et al., 2025). Baicalein/chitosan-modified zinc oxide nanoparticles (ZnO@CSbaicalein/Au NPs) were prepared by loading baicalein on chitosan-modified zinc oxide, which inhibited the proliferation of gastrointestinal stromal tumor cells by regulating PI3K/Akt/mTOR pathway and cell cycle-related gene expression (Ye et al., 2025). In addition, baicalein was encapsulated in tyrosine (Tyr)-HA-β-cyclodextrin grafted chitosan (CD-CS) carrier to prepare Tyr/HA/CD-CS-baicalein nano-delivery system. Relying on the bacterial targeting of HA and the biofilm dispersion of D-tyrosine, the Tyr/HA/CD-CS-baicalein nano-delivery system enhanced the permeability and bactericidal ability of drugs to *S. aureus* biofilms, and achieved efficient biofilm elimination (Tang L. et al., 2024). In addition, the BGZ@GelMA injectable photocrosslinked hydrogel was prepared by embedding baicalein and glucose oxidase (GOx) into ZIF-8 nanoparticles and then embedding methacrylated gelatin (GelMA). The BGZ@GelMA injectable photocrosslinked hydrogel can consume local glucose of diabetic wounds and reduce pH to regulate the microenvironment. At the same time, it can restore mitochondrial homeostasis by activating PPAR signaling pathway, synergistically release Zn^2+^ and baicalein to exert antibacterial and anti-inflammatory effects and promote wound healing (Qin et al., 2025).

These nanoformulations leverage carrier-specific targeting, enzyme-mimetic activity, and controlled-release properties to address the low solubility and poor bioavailability of flavonoids. By enabling precise regulation for different diseases, they highlight the multi-domain application potential of flavonoid nanoformulations.

### 9.2 Liposomes

Liposomes are microvesicles formed by encapsulating drugs within lipid bilayers, offering low toxicity, targeted delivery, and enhanced drug bioavailability (Fidan et al., 2024).

**FIGURE 10:**
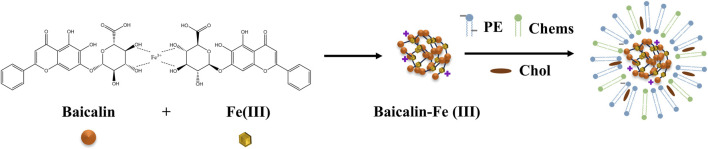
Baicalin-Fe (III) coordination-polymerloaded liposome.

**FIGURE 11:**
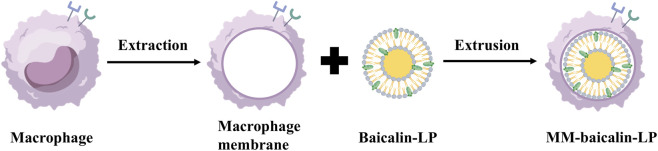
MM-baicalin-LP.

Chai et al. (2024) utilized coordination chemistry to combine baicalin with Fe (III), forming a baicalin-Fe (III) coordination-polymer-loaded liposome. This liposome, prepared by electrostatic adsorption with phosphatidylethanolamine, cholesterol, and cholesteryl hemisuccinate, was designed for the treatment of triple-negative breast cancer. The apoptosis rate of tumor cells treated with this liposome was 67.63% ± 10.48%, which was significantly higher than that of the free baicalin group (59.32% ± 5.75%). This result indicates that the baicalin-Fe (III) coordination-polymerloaded liposome has a significant impact on inducing apoptosis in triple-negative breast cancer cells and enhances the targeting ability of tumor sites (Figure 10). Building on the characteristic ability of borneol to facilitate drug passage through the blood-brain barrier, Long et al. (2023) investigated the effect of borneol-baicalin liposomes on enhancing brain targeting. *In vivo* studies showed that borneol in these liposomes promoted baicalin’s ability to cross the blood-brain barrier, thereby improving the brain-targeting efficiency of the liposomes. This resulted in superior therapeutic effects on cerebral ischemia-reperfusion injury in mice compared to baicalin liposomes alone. Moreover, borneol-baicalin liposomes facilitated the efficient delivery of baicalin to brain tissue through the downregulation of the HIF-1α/VEGF/endothelial nitric oxide synthase (eNOS)/nitric oxide (NO) pathway. This mechanism enhanced baicalin concentrations in both plasma and brain tissue in normal mice as well as those with cerebral ischemia-reperfusion injury. Further studies by Long et al. (2022) demonstrated that macrophage membrane modified baicalin liposome (MM-baicalin-LP) exhibited enhanced brain targeting capabilities, as confirmed by fluorescence imaging (Figure 11). At the same dose, the macrophage membrane-modified baicalin liposome group (2.00 ± 0.75) showed significantly better nerve improvement in MCAO model rats compared to the baicalin liposome group (2.50 ± 1.00). Additionally, the ischemic rate in the macrophage-modified liposome group (18.09 ± 4.56) was lower than that in the baicalin liposome group (12.01 ± 3.29), and the area of cerebral infarction in rats was reduced. These results suggest that macrophage membrane-modified baicalin liposomes can significantly mitigate cerebral ischemia-reperfusion injury. The long-circulating liposomes of baicalein were prepared by thin film hydration and modified by DSPE-PEG2000 to prolong the circulation time *in vivo*. The encapsulation efficiency was about 87.98% and the drug loading was about 7.56%. In the mouse model of traumatic brain injury (TBI), it can increase the distribution of baicalein in the brain, improve the pathological damage and neurological motor function of brain tissue, and reduce the release of inflammatory factors and neurotoxicity markers (LDH) (Yuan et al., 2024). HA and polyhexa methylene guanidine (PHMG) double-modified baicalein-loaded liposomes were also prepared by thin-film hydration. HA can target the CD44 receptor highly expressed at the site of infection. PHMG enhances the affinity of bacteria, and can respond to the acidic pH of the infection microenvironment and the lipase secreted by bacteria to achieve on-demand release of baicalein. In the mouse subcutaneous abscess and thigh muscle infection model induced by methicillin-resistant *S. aureus*, it can effectively inhibit planktonic bacteria and biofilms, reduce bacterial load and inflammatory infiltration at the site of infection. At the same time, it has good biocompatibility with low hemolysis and no obvious liver and kidney damage (Zang et al., 2024).

Baicalin liposomes also show promise in food preservation. Lu et al. (2022) utilized electrospinning technology to incorporate baicalin liposomes into polyvinyl alcohol-chitosan substrates, creating nanofibrous films. These films were found to transfer baicalin to bacterial cell membranes, causing bacterial deformation or destruction. The films exhibited inhibitory effects on *Escherichia coli* and *S. aureus* without cytotoxicity. Additionally, these baicalin-loaded films prevented browning and rancidity in mushrooms, offering a potential application in food preservation packaging.

### 9.3 Solid dispersions

**FIGURE 12:**
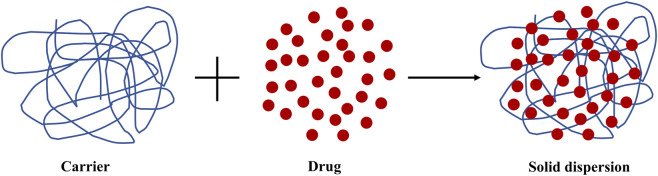
Schematic diagram of solid dispersions.

Solid dispersion is a kind of dispersion system in the form of solid, which is formed by uniformly dispersing the drug in the carrier in a highly dispersed state such as molecule, amorphous and microcrystalline forms (Figure 12) (Patel et al., 2022). Solid dispersions can be categorized into four types based on molecular arrangement: eutectic mixtures, solid solutions, amorphous solid solutions, and glass solutions and glass dispersions. They can also be classified according to the type of carrier used: crystalline carriers, polymer carriers, surfactant and polymer mixture carriers, and water-insoluble polymer carriers (Cid et al., 2019).

Studies have shown that the solubility and dissolution rate of three main flavonoids in SR root extract-baicalein, wogonin and oroxylin A were significantly improved by solid dispersion technology using hydrophilic polymer PVP K-30 as a carrier (Yu et al., 2017). Yang A. X. et al. (2020) prepared a baicalin-lecithin phospholipid complex and subsequently formed a baicalin phospholipid complex solid dispersion with PVP-K30. Pharmacokinetic studies showed that after baicalin was formulated into the phospholipid complex, the peak concentration (C_max_) increased by 1.979 times compared to the raw material. When baicalin was further formulated into a solid dispersion, C_max_ was increased by 2.634 times. The AUC_0-t_ of baicalin was 7.673 μg·h·ml^−1^, while the AUC_0-t_ of the baicalin phospholipid complex increased to 20.188 μg·h·ml^−1^, indicating a 263% improvement in relative bioavailability. After baicalin was formulated into a solid dispersion, AUC_0-t_ increased to 27.182 μg·h·ml^−1^, and the relative bioavailability improved to 354%. These results showed that baicalin phospholipid complex solid dispersion significantly enhances the oral bioavailability of baicalin compared to the phospholipid complex alone. Zhao et al. (2020) also prepared baicalin solid dispersions with PVP-K30 and used the extrusion-spheronization method to form pellets, which were tested in an *in vitro* dissolution simulation of the intestinal fluid environment. The release curve showed that after 10 min, the cumulative release of the raw material group reached nearly 96%, while the solid dispersion group showed only 12%, and the pellet group reached 60%. After 30 min, the cumulative release of both the pellet and raw material groups approached 100%, while the solid dispersion group reached 75%. This indicates that the baicalin solid dispersion and pellets can reduce baicalin’s solubility in weakly alkaline environments, thereby minimizing the first-pass effect in the body and enhancing its therapeutic efficacy. Yan and Qi (Yan and Qi, 2020) pioneered the use of polyethylene glycol 6000 (PEG6000) and polyethylene oxide (PEO) as carriers for solid dispersion, specifically focusing on the optimal preparation method for sustained-release baicalin solid dispersion by evaluating *in vitro* dissolution profiles. The study found that the highest cumulative dissolution rate occurred when baicalin was formulated into a sustained-release solid dispersion using a 1:1:3 ratio of baicalin to PEO and PEG6000, resulting in approximately 90% of the drug being released within 8 h. In this formulation, baicalin was in an amorphous form, and the sustained-release solid dispersion exhibited slow, controlled drug release, reducing the frequency of daily doses.

The carrier materials used in solid dispersions are typically hydrophilic, which helps increase the solubility of poorly soluble drugs and enhance their bioavailability.

### 9.4 Metal complexes

**FIGURE 13:**
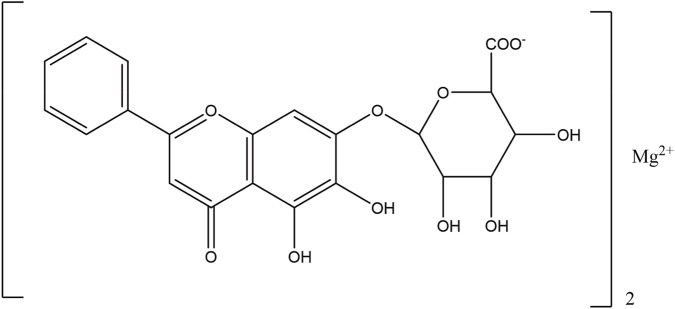
Structure of baicalin magnesium salt.

The molecular structure of baicalin contains hydroxyl and carbonyl groups, which confer a strong chelating ability with metal ions. Studies have shown that the original form of baicalin in SR is magnesium salt (Figure 13). The solubility of baicalin magnesium salt (129.1 mg/mL) is 2225 times greater than that of baicalin (0.058 mg/mL), making it highly soluble in water and easily extractable through water dissolution (Xu et al., 2017).

In a study by Zhang et al. (2024), the water extract of SR was recrystallized after alcohol precipitation to obtain baicalin with improved solubility. The conjugated form of baicalin with magnesium (baicalin-Mg) was characterized using UV, IR, atomic absorption spectroscopy, elemental analysis, ^1^H NMR, ^13^C NMR, and ESI-HRMS. The effect of baicalin-Mg on ulcerative colitis was explored in a mouse model. The results indicated that baicalin-Mg, at an equimolar concentration, exhibited superior effects compared to baicalin alone. baicalin-Mg significantly inhibited oxidative stress induced by dextran sodium sulfate and reduced the inflammatory response in dextran sodium sulfate-stimulated ulcerative colitis. Guan et al. (2023) established a rat model of nonalcoholic steatohepatitis through a high-fat diet and administered baicalin magnesium at a dose of 150 mg/kg. The treatment significantly mitigated lipid accumulation, inflammation, and oxidative stress induced by the high-fat diet, demonstrating a protective effect against nonalcoholic steatohepatitis in rats. In a mouse model of acute lung injury induced by LPS, Zhang L. et al. (2021) found that intraperitoneal injection of baicalin-Mg, baicalin, and MgSO_4_ significantly enhanced antioxidant capacity and reduced inflammatory cell infiltration in the lungs. The therapeutic effect of baicalin-Mg was notably superior to that of baicalin and MgSO_4_, highlighting its potential for treating acute lung injury.

**FIGURE 14:**
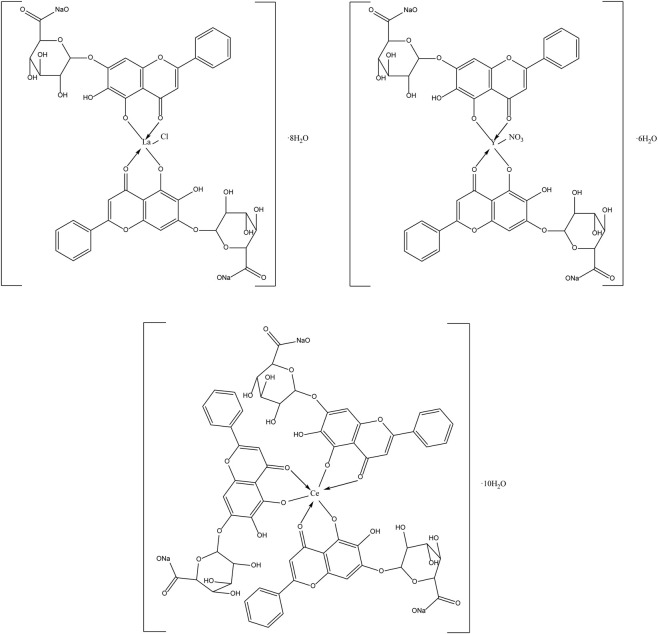
Structure of baicalin-La, baicalin-Y, baicalin-Ce.

**FIGURE 15:**
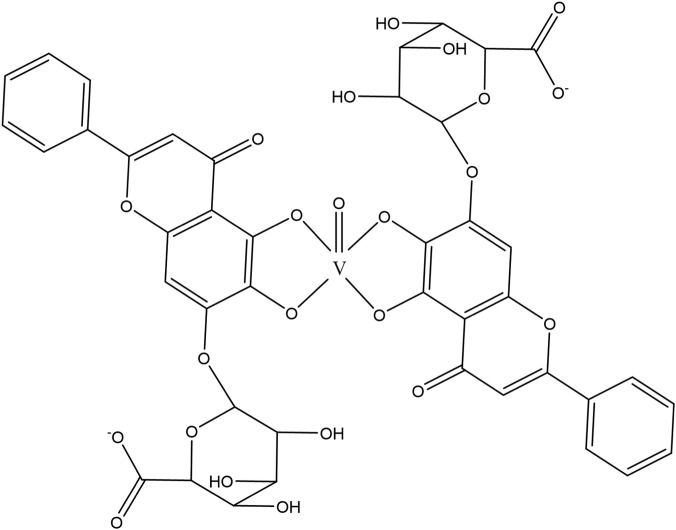
Structure of the complex formed by baicalin and oxidovanadium (IV).

When baicalin functions as a ligand, it can form complexes with various metal ions, including cerium, lanthanum, yttrium, and magnesium, enhancing its therapeutic efficacy. These baicalin-metal complexes are primarily coordinated with metal ions using the 5-hydroxyl and 4-carbonyl groups as binding sites (Li W. et al., 2023). Guo et al. (2020) synthesized three baicalin-metal complexes: cerium (III) [baicalin-Ce], lanthanum (III) [baicalin-La], and yttrium (III) [baicalin-Y] by complexing baicalin with rare earth metal ions (Figure 14). The composition and structure of the complexes were characterized through elemental analysis, thermogravimetric analysis, and UV and infrared spectroscopy. The inhibitory effects of these baicalin-metal complexes on the proliferation of SMMC-7721 cells were evaluated using a methyl thiazolyl tetrazolium assay. The results showed that baicalin, baicalin-Ce, baicalin-La, and baicalin-Y all exhibited significant effects after 72 h of treatment, with proliferation inhibition rates of 52.11%, 92.12%, 86.65%, and 82.79%, respectively. The order of antitumor activity was found to be baicalin-Ce>baicalin-La>baicalin-Y> baicalin. Additionally, Martínez Medina et al. (2017) studied the complexation of baicalin with oxidovanadium (IV) (Figure 15). The results demonstrated that this complex reduced the viability of the human lung cancer cell line A549, and the complexation with oxidovanadium enhanced the antioxidant capacity. The findings suggest that baicalin-metal complexes can arrest the cell cycle in the G2/M phase, upregulate Bax expression, downregulate Bcl-2 expression, and induce tumor apoptosis in a dose-dependent manner. The inhibitory effects on cancer cells were significantly superior to those of baicalin alone.

Baicalin magnesium salt optimizes baicalin’s physical and chemical properties through structural modification, improving solubility and bioavailability, reducing formulation development challenges, and expanding clinical administration routes.

In summary, research into new dosage forms of flavonoids is extensive, including nano-preparations, liposomes, solid dispersions, and metal complexes, in addition to double-network hydrogels (Wang et al., 2025), pH-responsive hydrogels (Lu et al., 2025), and porous silk fibroin microspheres (Gan et al., 2025). These new formulations have significantly enhanced the solubility, stability, bioavailability, and therapeutic effects of flavonoids. They facilitate targeted drug delivery, improving disease treatment outcomes and offering promising clinical applications (Table 8). However, it must be clearly recognized that most of the current studies are still in the preclinical stage, and their clinical transformation faces systematic challenges: not only the lack of approved clinical dosage forms, but also the lack of key data on long-term toxicity, human pharmacokinetics of preparations, and stability of large-scale production. Based on the existing pharmacodynamic and pharmacokinetic evidence, future transformation research should be focused on. Baicalin and baicalein can be regarded as candidate molecules for preferential transformation due to their clear anti-inflammatory, antiviral and anti-tumor activities and relatively clear metabolic pathways. In particular, baicalein, as an active aglycone, has become an ideal model compound for new preparations (such as liposomes and nano-preparations) due to the contradiction between its strong properties and low bioavailability.

**TABLE 8:** New dosage forms of flavonoids.

Dosage form	Compound type	Effects	References
Nano formulations	Baicalin, baicalein	Enhance tumor targeting and antibacterial/anti-inflammatory effects. Improve the low solubility and poor bioavailability of flavonoids.	Fan et al. (2024), Tian et al. (2023), Meher et al. (2023), Xiong et al. (2025), Ye et al. (2025), Tang et al. (2024b), Qin et al. (2025)
Liposomes	Baicalin, baicalein	Enhance solubility and stability, extend circulation time, and improve anti-tumor targeting.	Chai et al. (2024), Long et al. (2023), Long et al. (2022), Yuan et al. (2024), Zang et al. (2024), Lu et al. (2022)
Solid dispersions	Baicalin, baicalein, wogonin and oroxylin A	Using hydrophilic substances as carriers enhances the solubility of flavonoid compounds and improves their bioavailability, while also achieving a sustained-release effect.	Yu et al. (2017), Yang F. et al. (2020), Zhao et al. (2020), Yan and Qi (2020)
Metal complexes	Baicalin	Structural modifications were made to baicalin to optimize its physicochemical properties, thereby enhancing its solubility and bioavailability.	Xu et al. (2017), Zhang et al. (2024), Guan et al. (2023), Zhang L. et al. (2021), Li et al. (2023d), Guo et al. (2020), Martínez Medina et al. (2017)

From the perspective of application, in addition to solving scientific problems, it is also necessary to face the obstacles of supervision and industrialization to bridge the gap from laboratory to clinical practice. This includes: the quality control challenges brought by the complexity of preparations, and how to ensure the stability and consistency between batches of complex products such as nano-preparations; the safety evaluation system that meets the requirements of drug registration needs to carry out systematic non-clinical studies including immunotoxicity and reproductive toxicity. In the formulation of clinical development strategies, it is necessary to clarify the indications, dose exploration and biomarkers. Only by facing up to and systematically studying these obstacles can the innovative preparations of flavonoids move from active academic research to drugs that truly meet clinical needs.

Therefore, future research work should adhere to the transformation orientation, deepen the exploration of the mechanism, and focus on filling the above-mentioned transformation research gaps, so as to promote the substantive transformation of this field from discovery to development.

## 10 Conclusion

As an important component of traditional Chinese medicine SR, flavonoids have gained significant momentum in research into their *in vivo* metabolism, immunological and pharmacological effects, and various dosage forms, thanks to the continuous advancement of detection technologies such as ultra-high-performance liquid chromatography, mass spectrometry, nuclear magnetic resonance, and emerging fields like molecular biology, genomics, proteomics, and metabolomics. These efforts have led to key findings that provide a solid theoretical foundation for the development of new drugs and the clinical application of flavonoids. In the current study, baicalin and baicalein are the most promising flavonoids, which show clear pharmacological activities in the fields of anti-inflammatory, anti-tumor and anti-virus. The nano-preparations, liposomes, solid dispersions, and metal complex preparations of flavonoids have effectively improved bioavailability by improving drug solubility, prolonging *in vivo* circulation time, and achieving targeted delivery. It has become the focus of preparation research and development and provides a clear candidate target for subsequent clinical research. It needs to be clarified that most of the studies on flavonoids in SR are still in the preclinical stage, including *in vitro* cell experiments, animal model verification and partial pharmacological mechanism analysis. Although its potential medicinal value has been confirmed, there is still a gap from large-scale clinical application. At present, the core bottlenecks are still focused on: the low bioavailability caused by poor fat solubility and water solubility of some flavonoids, the balance between multi-effect and specificity of action targets, and the lack of drug safety data for special populations, which all limit its clinical transformation process. Future research on flavonoids should prioritize in-depth studies of its *in vivo* processes, detailed analysis of drug interactions, and the expansion of large-scale clinical trials. The efficacy and safety of flavonoids across different disease models must be validated in comprehensive clinical studies to fully realize its clinical potential.

In future studies on the mechanism of pharmacological action, the selection of drug doses and the optimization of therapeutic effects should prioritize minimizing side effects while maximizing efficacy. A comparative analysis of pharmacokinetic parameters (such as AUC, T_max_, and C_max_) across different doses should be conducted, and the dose and frequency of administration should be tailored to the specific disease model. Additionally, the optimal dose range of flavonoids in compound formulations can be explored through orthogonal experimental designs, aiming to balance efficacy with minimal toxicity. Currently, the safety data for flavonoids is primarily derived from short-term studies, necessitating the implementation of long-term toxicity research that focuses on liver and kidney function, immune responses, and gut microbiota. For special populations, including pregnant women, children, and individuals with liver or kidney dysfunction, risk evaluation should be carried out using animal models and clinical cohort studies, leading to the development of targeted dosing regimens. Concurrently, establishing an adverse reaction monitoring network and leveraging big data analysis to identify potential risk signals, such as allergic reactions and cardiovascular events, will be essential for developing predictive models for risk warning. To address the challenge of flavonoids’ efficacy under various pathological conditions, precise pathological models should be developed, focusing on the mechanisms and key targets using multi-omics analysis. Innovations in dosage forms and individualized treatment strategies are necessary to overcome the limitations of low bioavailability and unclear targets, paving the way for clinical application.

As a bridge between traditional medicine and modern pharmacology, flavonoids demonstrate the potential of natural products in drug discovery. Future research should integrate omics technologies and combinatorial therapies to fully unlock its clinical potential, ultimately advancing personalized and sustainable healthcare solutions.
